# Genome-wide analysis of *Brucella melitensis* genes required throughout intranasal infection in mice

**DOI:** 10.1371/journal.ppat.1010621

**Published:** 2022-06-30

**Authors:** Georges Potemberg, Aurore Demars, Emeline Barbieux, Angéline Reboul, François-Xavier Stubbe, Malissia Galia, Maxime Lagneaux, Audrey Comein, Olivier Denis, David Pérez-Morga, Jean-Marie Vanderwinden, Xavier De Bolle, Eric Muraille

**Affiliations:** 1 Unité de Recherche en Biologie des Microorganismes (URBM)—Laboratoire d’Immunologie et de Microbiologie, NARILIS, University of Namur, Namur, Belgium; 2 Laboratoire de Parasitologie, and ULB Center for Research in Immunology (U-CRI), Université Libre de Bruxelles, Gosselies, Belgium; 3 Unité de recherche en physiologie moléculaire (URPhyM)—Laboratoire de Génétique moléculaire (GéMo), University of Namur, Namur, Belgium; 4 Immunology Unit, Scientific Institute for Public Health (WIV-ISP), Brussels, Belgium; 5 Center for Microscopy and Molecular Imaging, Université Libre de Bruxelles (ULB), Gosselies, Belgium; 6 Laboratory of Neurophysiology, Université Libre de Bruxelles, Campus Erasme, Brussels, Belgium; Tufts University, UNITED STATES

## Abstract

*Brucellae* are facultative intracellular Gram-negative coccobacilli that chronically infect various mammals and cause brucellosis. Human brucellosis is among the most common bacterial zoonoses and the vast majority of cases are attributed to *B*. *melitensis*. Using transposon sequencing (Tn-seq) analysis, we showed that among 3369 predicted genes of the *B*. *melitensis* genome, 861 are required for optimal growth in rich medium and 186 additional genes appeared necessary for survival of *B*. *melitensis* in RAW 264.7 macrophages *in vitro*. As the mucosal immune system represents the first defense against *Brucella* infection, we investigated the early phase of pulmonary infection in mice. *In situ* analysis at the single cell level indicates a succession of killing and growth phases, followed by heterogenous proliferation of *B*. *melitensis* in alveolar macrophages during the first 48 hours of infection. Tn-seq analysis identified 94 additional genes that are required for survival in the lung at 48 hours post infection. Among them, 42 genes are common to RAW 264.7 macrophages and the lung conditions, including the T4SS and purine synthesis genes. But 52 genes are not identified in RAW 264.7 macrophages, including genes implicated in lipopolysaccharide (LPS) biosynthesis, methionine transport, tryptophan synthesis as well as fatty acid and carbohydrate metabolism. Interestingly, genes implicated in LPS synthesis and β oxidation of fatty acids are no longer required in Interleukin (IL)-17RA^-/-^ mice and asthmatic mice, respectively. This demonstrates that the immune status determines which genes are required for optimal survival and growth of *B*. *melitensis in vivo*.

## Author summary

Brucellosis is one of the most widespread bacterial zoonoses worldwide. Using transposon sequencing (Tn-seq) analysis, we showed that among 3369 predicted genes of the *Brucella melitensis* genome, 861 are required for optimal growth in rich medium and 186 additional genes appeared necessary for survival of *B*. *melitensis* in RAW 264.7 macrophages *in vitro*. We also investigated the early phase of pulmonary infection in mice and identified 94 additional genes that are required for survival in the lung at 48 hours post infection. Among them, 42 genes are common to RAW 264.7 macrophages and the lung conditions, including the T4SS and purine synthesis genes. But 52 genes are not identified in RAW 264.7 macrophages, including genes implicated in lipopolysaccharide (LPS) biosynthesis, methionine transport, tryptophan synthesis as well as fatty acid and carbohydrate metabolism. Interestingly, genes implicated in LPS synthesis and β oxidation of fatty acids are no longer required in Interleukin (IL)-17RA^-/-^ mice and asthmatic mice, respectively. Our work demonstrates that both the immune status and the nature of the infected cell type determines which genes are required for optimal survival and growth of *B*. *melitensis in vivo*.

## Introduction

*Brucellae* are small Gram-negative facultative intracellular bacteria which belong to the Rhizobiales order within the α2-proteobacteria subgroup. They are the causative agent of brucellosis, one of the most common bacterial anthropozoonoses that generates major economic losses and public health issues. *B*. *melitensis* is the species most often involved in ovine and caprine brucellosis and is also the most pathogenic species for humans [1]. Human brucellosis primarily occurs following ingestion of contaminated foods or mucosal exposure to contaminated aerosols [2][3][4]. It is characterized by nonspecific flu-like symptoms during the early acute phase, and is followed by a chronic infection with debilitating consequences in the absence of prolonged antibiotic treatment [1][5].

During the host infection, *B*. *melitensis* mainly leads a stealthy intracellular lifestyle [6]. The type IV secretion system (T4SS), which is encoded by the *virB* operon, is required for the establishment of intracellular replicative niches [7]. *B*. *melitensis* strains lacking a functional T4SS appear to be highly attenuated in mice and in their natural host, the goat [8]. Over the last decade, our group has characterized the protective response against *B*. *melitensis* in an intranasal (i.n.)[9][10] murine infection model and demonstrated that the early phase of lung infection is controlled by an Interleukin (IL)-17RA-dependent Th17 response and the late phase by an IFN-γR-dependent Th1 response. We also demonstrated that the asthma-induced Th2 response can greatly increase the *Brucella* load in the lungs [11].

Signature-tagged mutagenesis (STM)[12] or more classical screening of transposon mutants has been employed to identify genes that are essential for *Brucella* growth and survival in a macrophage cell line *in vitro* [13][14], in mice [15][16][17] and in natural hosts such as the goat [18]. However, STM uses a limited number of transposon mutants for screening, thus failing to truly saturate the entire genome. Transposon sequencing (**Tn-seq**) is a recent powerful approach to rapidly and comprehensively determine an organism’s minimal genetic requirements for growth and survival under a variety of different conditions [19][20]. A high-density transposon insertion library is exposed to a condition of interest, and then subjected to high-throughput sequencing to map the transposon insertion site for each mutant in the library. The number of reads detected for each insertion mutant is proportional to the fitness of that mutant under the selected growth condition. Recently, a Tn-seq screen of *B*. *abortus* identified many genes important for growth in a nutrient rich media and *in vitro* in RAW 264.7 macrophages [21]. However, much remains to be learned regarding genes required for the survival of *Brucella* in a well characterized *in vivo* host infection model.

In the current study, we characterized the initial growth of *B*. *melitensis* in the lungs of intranasally-infected C57BL/6 mice in order to identify the early selection pressures affecting its multiplication. Then, we used Tn-seq screens to compare genes contributing to the fitness of *Brucella melitensis* in 2YT nutrient rich media, in RAW 264.7 macrophages and in lungs from wild-type C57BL/6 mice. To identify genes specifically implicated in the escape from the immune response, we also performed Tn-seq screens on infected mice deficient for the control of *Brucella* growth, such as IL-17RA^-/-^ [9] and asthmatic mice [11].

## Results

### Comparison of *B*. *melitensis* multiplication in a RAW 264.7 cell line and the lung

Our main objective was to use a Tn-seq approach to identify bacterial genes essential for the initiation of *B*. *melitensis* infection *in vivo*. To produce infection by a natural route that confronts *B*. *melitensis* with mucosal immunity, we chose a well characterized model of intranasal (i.n.) infection in the mouse model [9] that mimics aerosol infection.

As shown previously [10], i.n. infection produces a specific pattern of bacterial dissemination, limited to a small number of tissues. *B*. *melitensis* must colonize the lung and the mediastinal lymph node before spreading and establishing in the spleen. To characterize the infected cells in the i.n. model, we used a mCherry-expressing stain of *B*. *melitensis* (mCherry-*Br*) stained with eFluor^670^ (eFluor), which is a dye used to monitor polar growth [10]. We observed that, during the first 48 hours post-infection, the eFluor^+^ lung cells from intranasally infected wild-type C57BL/6 mice are FSC^high^ (**S1A Fig**) MHCII^med^ F4/80^med^ CD11b^med^ CD11c^high^ Ly6C^low^ Ly6G^low^ Siglec-F^high^ (**S1B Fig**), thus demonstrating that the cells infected in the lung are mainly alveolar macrophages (AMs). We hypothesize that alveolar macrophages constitute one of the first lines of defense against pulmonary *B*. *melitensis* infection in our experimental model.

The Tn-seq approach is known to be highly sensitive to bottleneck effects [22]. Many false positive classifications are observed when stochastic loss of transposon mutants due to bottlenecks overshadows the effects of fitness defects. The risk of trans-complementation, where a strain with a transpositional (Tn) mutation is able to survive or multiply thanks to the presence of strains with a wild-type allele, resulting from coinfection of a single host cell with several independent Tn mutants, must also be reduced [23].

**Fig 1:**
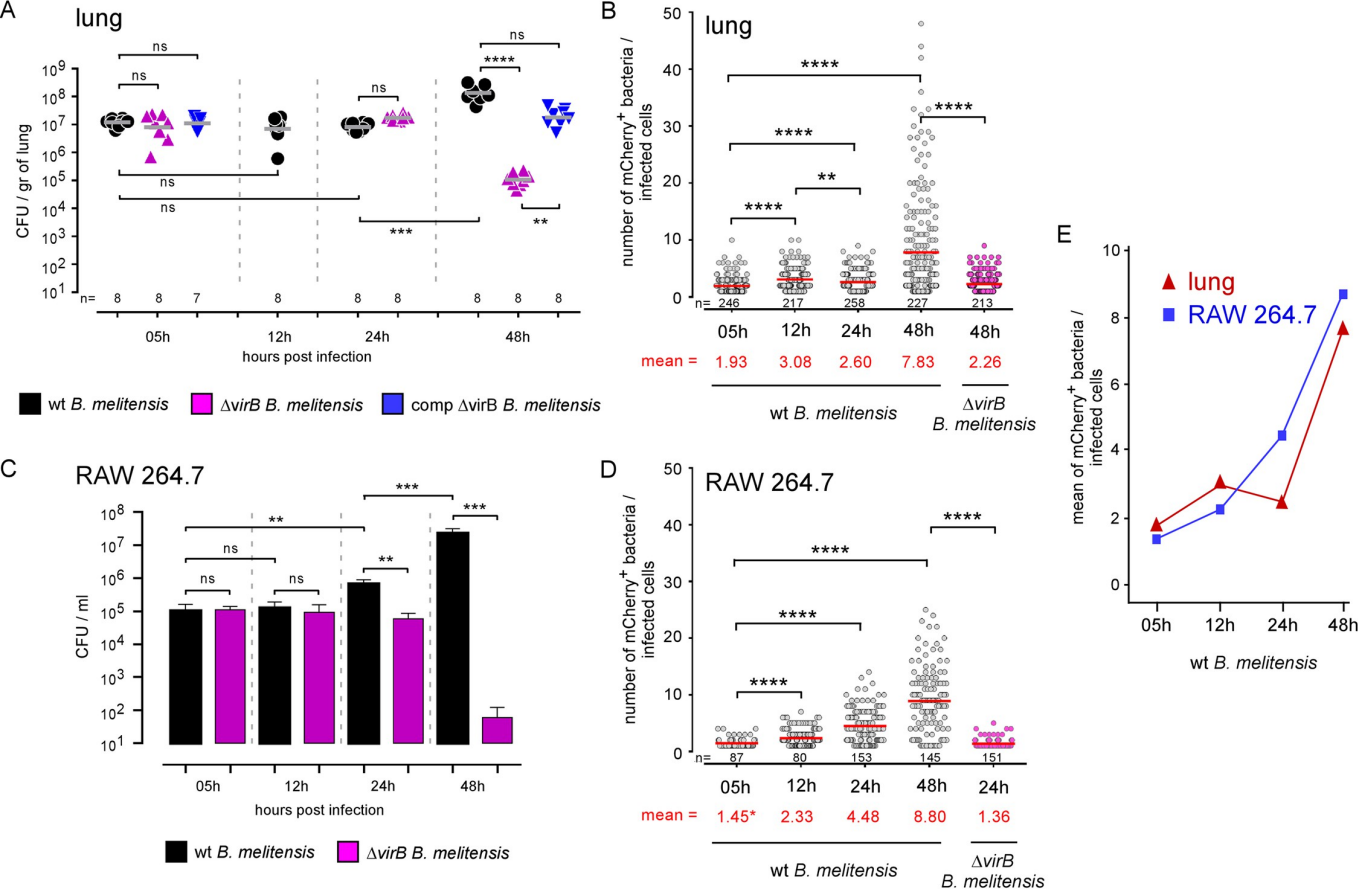
Comparison of *B*. *melitensis* multiplication in lungs of wild-type mice and RAW 264.7 macrophages. **A, B.** C57BL/6 mice (n = 7–8) were infected with 5x10^6^ CFU of mCherry-expressing *B*. *melitensis* and sacrificed at the indicated time. Lungs were harvested for CFU and fluorescent microscopy analysis. Data represent (**A**) the CFU count per g of lung from individual mice (n indicates the number of mice per group) and (**B**) the number of mCherry^+^ bacteria per cell determined by fluorescent microscopy (n indicates the number of infected cells observed per group). **C, D.** RAW 264.7 macrophages were infected with an MOI of 50 (50 bacteria per cell on average). Data shown are (**C**) the CFU count per condition and (**D**) the number of mCherry^+^ bacteria per infected cell (n indicates the number of infected cells observed per group). **E.** Data represent the comparison of the average number of mCherry^+^ bacteria per infected lung cell and per infected RAW 264.7 macrophage. Significant differences between the indicated groups are marked with asterisks: **p < 0.01, ***p < 0.001, ****p < 0.0001, in a One-Way ANOVA with Kruskal-Wallis post-test. CFU results (**A** and **C**) are representative of three independent experiments. Microscopy bacteria count data for lung (**B**) are pooled from 2 independent *in vivo* experiment. For each experiment, the lungs of 3 mice were analyzed by fluorescence microscopy. Microscopy bacteria count data for RAW 264.7 (**D**) are pooled from 2 independent experiments.

To avoid these problems, we used an infection dose that reduces the risk of bottleneck effects while limiting the number of bacteria initially phagocytosed per cell to one on average. Following infection with 5x10^6^ colony forming units (CFU) of wild-type mCherry-*Br*, the CFU count in the lungs of wild-type C57BL/6 mice remained stable at 10^7^ CFU/g, which is approximately half the infectious dose, between 5 and 24 hours post-infection, and then reaches 10^8^ CFU at 48 hours post-infection (**Fig 1A**). Fluorescent microscopic analysis of lung sections showed that the average number of mCherry^+^ bacteria per cell at 5 hours post-infection was 1.93 (**Fig 1B**). Similar experiments with Δ*vir*B mCherry-*Br* confirmed that *B*. *melitensis* persistence in the lungs (**Fig 1A**) and *B*. *melitensis* multiplication in AMs (**Fig 1B**) require a functional T4SS, like in RAW 264.7 macrophages, a cell line frequently used for the *in vitro* study of *Brucella* infection (**Fig 1C** and **1D**).

Interestingly, although the number of CFU in the lung remained stable between 5 and 24 hours post-infection (**Fig 1A**), we observed a first peak in the number of bacteria per cell at 12 hours and a second at 48 hours post-infection (**Fig 1B**), which suggests that there may be a selection step between 12 and 24 hours post-infection. This phenomenon was not observed in infected RAW 264.7 macrophages, where the number of CFUs (**Fig 1C**) as well as the number of bacteria per cell (**Fig 1D**) increased steadily between 5 and 48 hours post-infection (see **Fig 1E** for a comparison between the lung and RAW 264.7 macrophages).

### *B*. *melitensis* undergoes early selection pressures in the lung

In order to further analyze *B*. *melitensis* multiplication and survival at the single-cell level *in vivo* and confirm the existence of early selection of *B*. *melitensis*, we administrated intranasally 5x10^6^ CFU of mCherry-*Br* stained with eFluor in wild-type C57BL/6 mice and performed fluorescent and confocal microscopy analysis on lung sections. As shown previously [24][10], the unipolar growth of *B*. *melitensis* [25] allowed us to discriminate (**S2A–S2B Fig**) between non-growing mother cells (mCherry^+^ eFluor^+^), growing mother cells (mCherry^+^ eFluor^neg/+^, i.e. bacteria positive for eFluor staining on only a fraction of their surface), daughter cells (mCherry^+^ eFluor^neg^) and presumed dead mother cells (mCherry^neg^ eFluor^+^).

**Fig 2:**
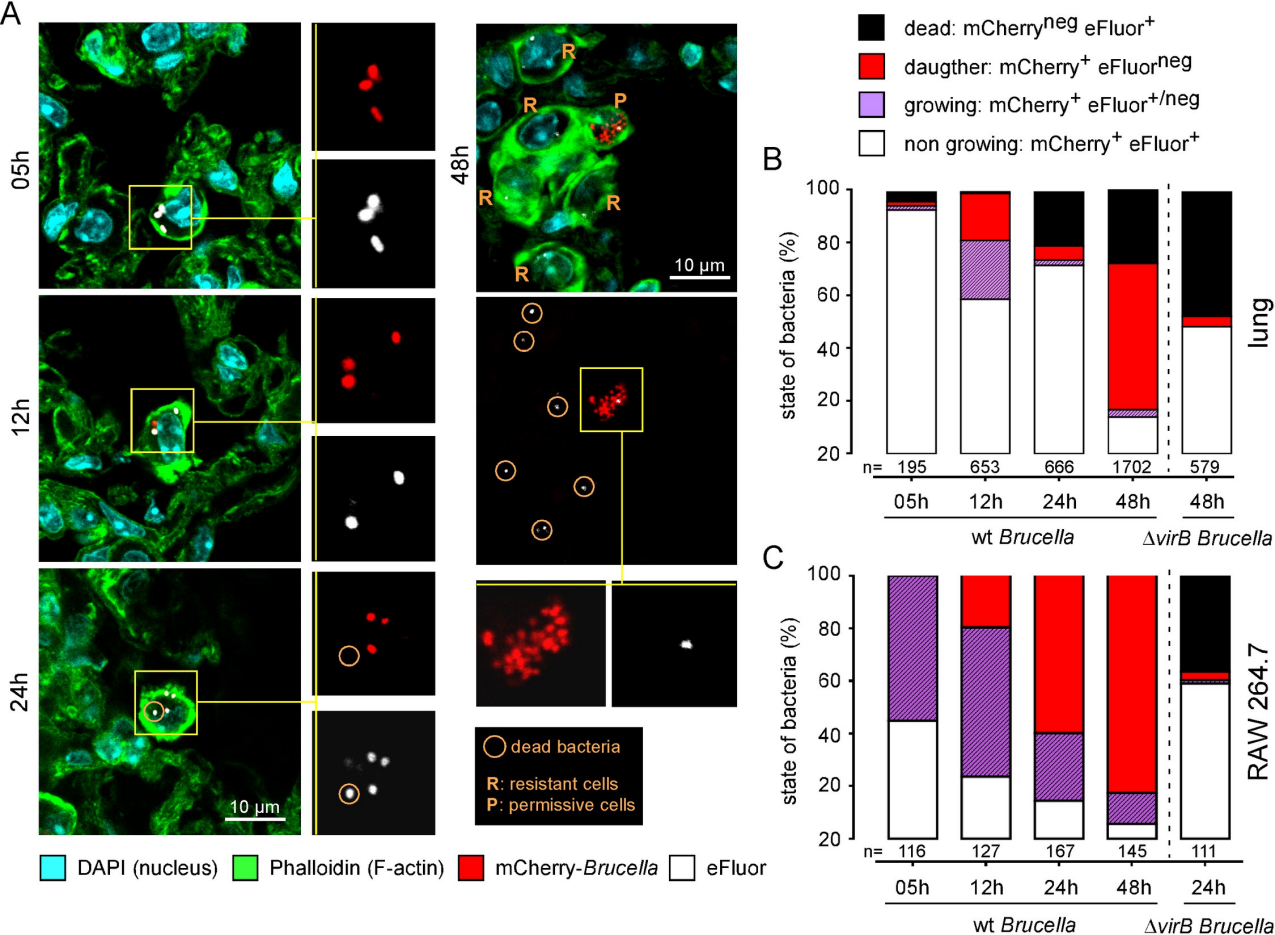
Fluorescent microscopic analysis of *B*. *melitensis* multiplication in lungs of wild-type mice and RAW 264.7 macrophages. **A, B.** C57BL/6 mice (n = 5) were infected with 5x10^6^ CFU of mCherry-expressing *B*. *melitensis* labelled with eFluor^670^ and sacrificed at the indicated time. Lungs were collected and analyzed by fluorescent and confocal microscopy for the expression of DAPI, phalloidin, mCherry and eFluor^670^. Data shown are (**A**) representative confocal images (single z-plane) of infected lungs and (**B**) the frequency of non-growing mother cells, growing mother cells, daughter cells and presumed dead mother cells estimated using a fluorescent microscope (n indicates the number of infected cells observed per group). **C.** RAW 264.7 macrophages were infected with an MOI of 50 (50 bacteria per cell on average) and analyzed by fluorescent microscopy at the indicated time. Data shown are (**C**) the frequency of non-growing mother cells, growing mother cells, daughter cells and presumed dead mother cells (n indicates the number of infected cells observed per group). These results are representative of two independent experiments.

**Fig 2A** shows representative confocal microscopy images from infected cells at 5, 12, 24 and 48 hours post-infection in the lung. A count of more than 100 cells per condition (**Fig 2B**) demonstrated that infected cells contain mainly non-growing bacteria at 5 hours post-infection and that rapid bacterial growth does not occur in AMs. At 12 hours post-infection, growing and daughter bacteria appeared and together accounted for 40% of the bacteria counted. In agreement with the drop in the number of mCherry^+^ bacteria per infected cell (**Fig 1B**), we observed a drop in the proportion of growing and daughter bacteria and the appearance of ≃20% of dead bacteria at 24 hours (**Fig 2B**), confirming that the bacteria undergo strong selection in AMs between 12 and 24 hours post-infection. Interestingly, the proportion of growing and daughter bacteria was dramatically decreased at 24 hours post-infection compared to 12 hours post-infection (**Fig 2B**), suggesting that killing is more effective for growing bacteria compared to mother bacteria. At 48 hours post-infection, more than 50% of the bacteria were daughter bacteria, which suggests that bacteria that survive selection between 12 and 24 hours post-infection then actively multiply. A similar analysis (**Fig 2C**) performed on infected RAW 264.7 macrophages showed very different kinetics of *B*. *melitensis* multiplication. In these cells, the bacteria grew quickly, the percentage of daughter bacteria steadily increased, and no dead bacteria were detected (**Fig 2C**). In this experiment, we used Δ*vir*B strain to demonstrate that RAW 264.7 macrophages are indeed capable of killing *B*. *melitensis* if it cannot deflect its vacuolar traffic using T4SS.

Our fluorescent tracers have some limitations for monitoring bacteria *in vivo*. The most obvious is that dead daughter bacteria (mCherry^neg^ eFluor^neg^) are no longer detectable and that stressed bacteria in poor condition or degraded bacteria are not identifiable. To further characterize the state of bacteria in AMs, we performed transmission electron microscopy (TEM) analyses of purified AMs from mice infected for 5, 12, 24 and 48 hours with 10^8^ CFU of wild-type or Δ*virB B*. *melitensis*. However, as previously shown in **Fig 1B**, with an infection dose of 5x10^6^ CFU, infected AMs contain on average only one bacterium per cell at 5 hours post infection. Thus, the probability of observing bacteria in a section plane by TEM is therefore very low. In order to increase this probability, we use a higher dose of bacteria, 10^8^ CFU, to infect mice for these experiments.

To obtain unequivocal reference images of living and dead *B*. *melitensis*, we incubated *B*. *melitensis in vitro* for 30 min at 80°C or in minimal (Plommet-erythritol) medium for 24 hours before performing the TEM analyses. Bacteria cultivated in minimal medium, under nutritional stress, exhibit slower growth when compared to bacteria cultivated in 2YT rich medium (**S3A Fig**). TEM analysis showed that bacteria cultivated in minimal medium present small low-density areas in the cytoplasm and that heat killed bacteria displayed very large low-density areas in the cytoplasm (**S3B Fig**). We therefore assigned these morphologies to a state of “stressed” or “dead”. It is important to note that the stressed morphology did not prevent the multiplication of *B*. *melitensis*. Indeed, we observed dividing bacteria in both rich and minimal Plommet medium (**S3C Fig**).

**Fig 3:**
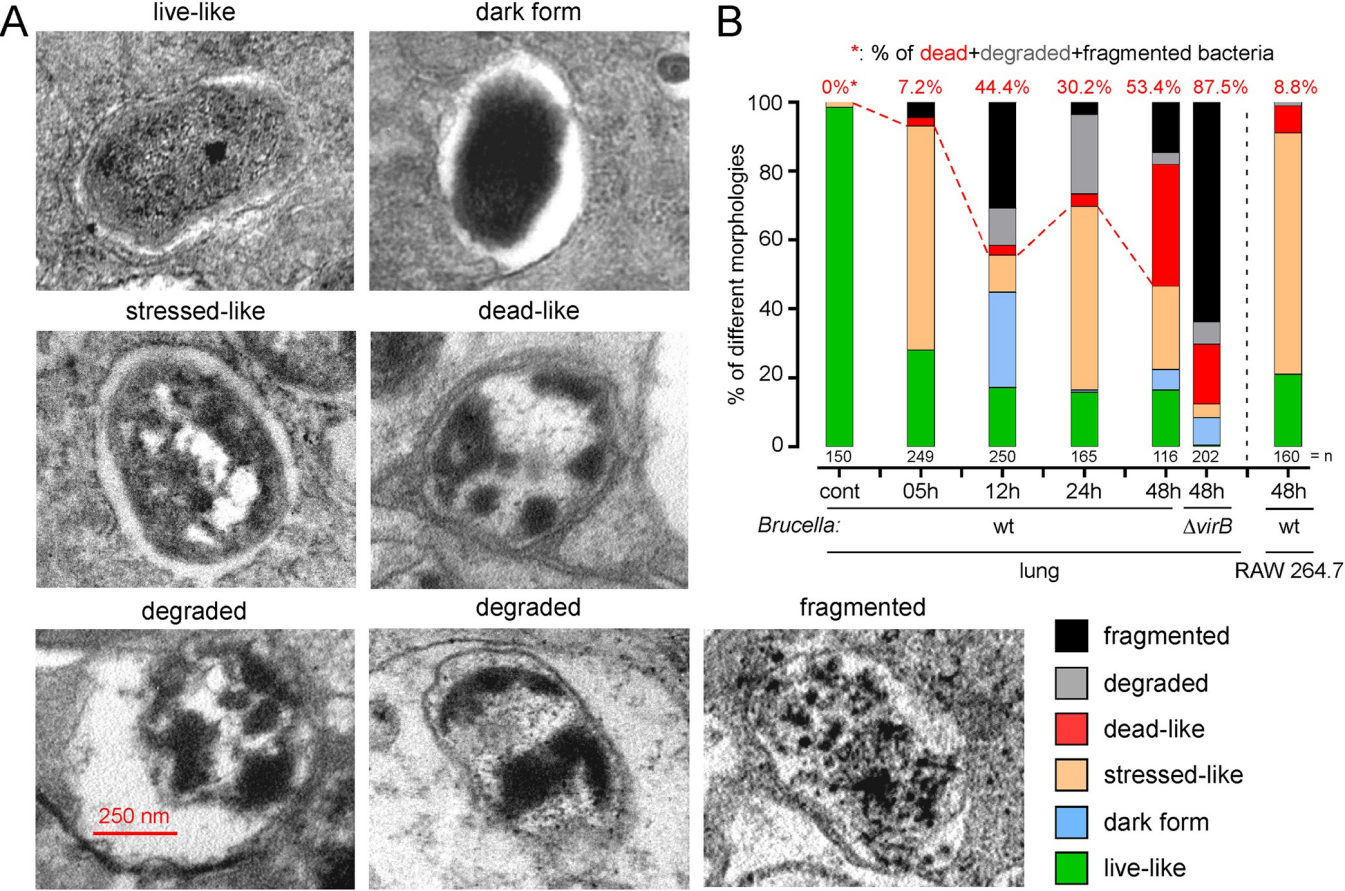
Transmission electron microscopic analysis of *B*. *melitensis* morphology inside alveolar macrophages purified from infected mice. C57BL/6 mice (n = 10) received intranasally PBS (uninfected mice) or 10^8^ CFU of mCherry-expressing wild-type or ΔvirB strains of *B*. *melitensis* and were sacrificed at the indicated time. Lungs were harvested at the indicated time (5, 12, 24, 48 hours post-infection) and alveolar macrophages were purified and analyzed by transmission electron microscopy as described in the Materials and Methods. RAW 264.7 macrophages were infected with an MOI of 50 (50 bacteria per cell on average) of an mCherry-expressing wild-type strain of *B*. *melitensis*, harvested at 48 hours post-infection and analyzed by transmission electron microscopy. Data shown are (**A**) representative images of bacteria from alveolar macrophages displaying live-like, dark, stressed-like, dead-like, degraded and fragmented morphologies, (**B**) the frequency of each bacterial morphology observed in each condition (n indicates the number of infected cells observed per condition). These results are representative of two independent experiments.

In AMs purified from infected mice, TEM analysis showed six distinct forms (called live-like, dark, stressed-like, dead-like, degraded and fragmented) of bacteria (**Fig 3A**). None of these forms were detected in AMs from uninfected mice. We measured the frequency of each form of bacteria before i.n. infection and for all *in vivo* conditions (5, 12, 24, 48 hours post-infection) (**Fig 3B**). Interestingly, over 50% of the bacteria already had a stressed-like morphology at 5 hours post-infection, suggesting that the bacteria were already undergoing environmental stress at this very early stage of infection. The proportion of dead-looking bacteria (including dead-like, degraded and fragmented forms) inside AMs gradually increased over time, from 7% at 5 hours post infection to 53% at 48 hours post infection. These percentages are very different from those obtained by fluorescence microscopy (**Fig 2A**). This is probably due to the fact that we do not detect dead daughter bacteria by fluorescence. The fact that the percentage of dead bacteria is not cumulative over time probably stems from the fact that the bacteria are no longer detectable at some extreme stage of degradation. At 48 hours post-infection, only 16% of wild-type bacteria display the live-like morphology. As expected, we only observed 0.4% of live-like bacteria inside AMs from mice infected for 48 hours with the Δ*vir*B strain.

Overall, our data demonstrate that *B*. *melitensis* is subjected in the lung to a more intense selection pressure than that present *in vitro* in macrophages RAW 264.7.

### *B*. *melitensis* multiplies exponentially in only a fraction of alveolar macrophages

Confocal microscopy of lungs infected for 48 hours (**Fig 2A**) showed that only a small fraction of infected cells was “permissive” to *Brucella* multiplication and contained a high number of daughter bacteria. At that point, most infected cells contain only dead bacteria and were considered as “resistant cells”.

**Fig 4:**
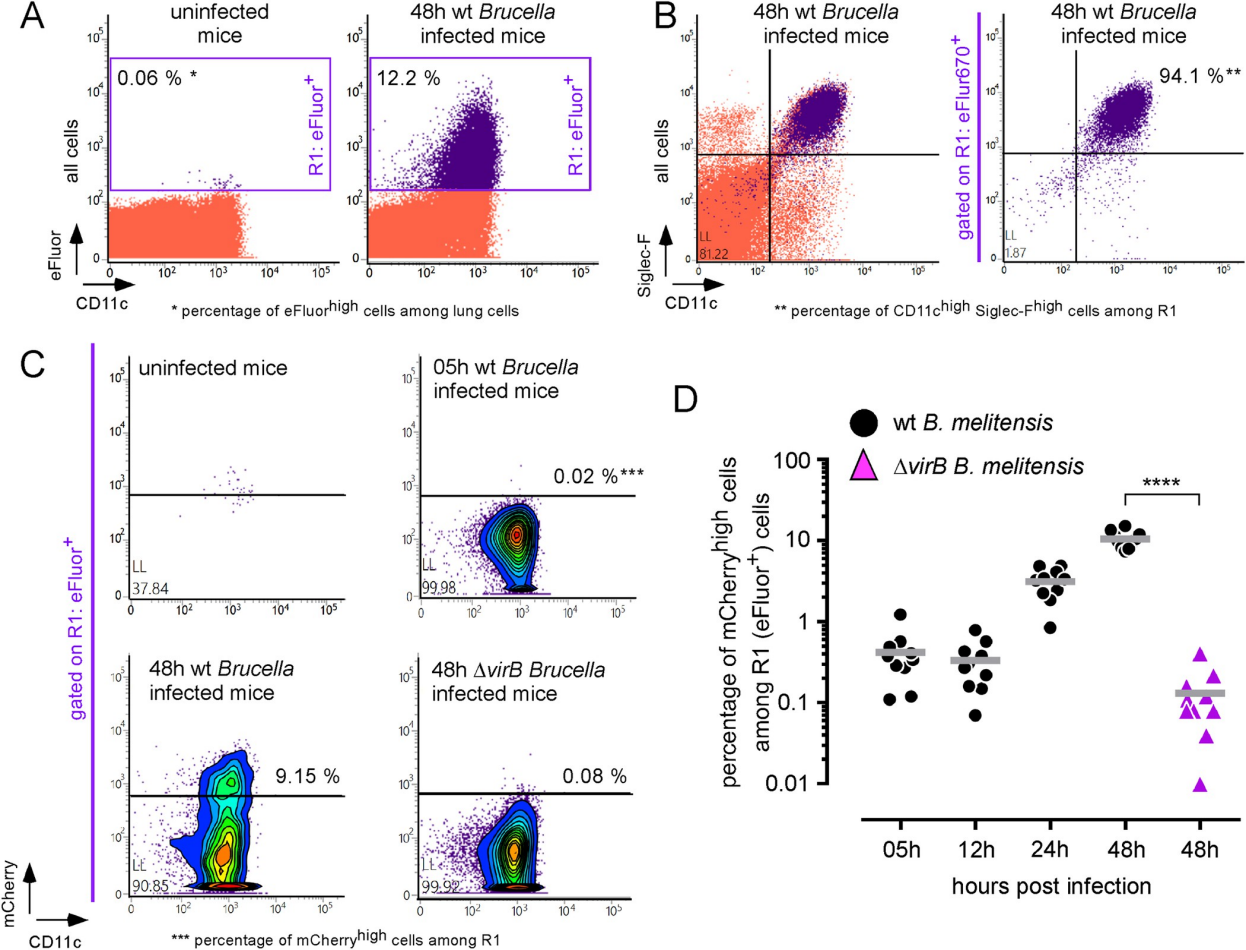
*B*. *melitensis* multiplies exponentially only in a fraction of alveolar macrophages. C57BL/6 mice (n = 10) received intranasally PBS (uninfected mice) or 5x10^6^ CFU of mCherry-expressing wild-type or ΔvirB strains of *B*. *melitensis* labelled with eFluor^670^. Mice were sacrificed at the indicated time. Lungs were collected and analyzed individually by flow cytometry for the expression of CD11c, Siglec-F, mCherry and eFluor^670^. Data shown are (**A**) representative dot plots of total lung cells from control and infected mice analyzed for the expression of CD11c and eFluor^670^, (**B**) representative dot plots of total lung cells and eFluor^+^ cells (R1 gate) analyzed for the expression of CD11c and Siglec-F, (**C**) representative dot plot of eFluor^+^ cells analyzed for the expression of CD11c and mCherry, (**D**) the kinetic percentage of mCherry^high^ cells among eFluor^+^ lung cells per individual mice (n = 10). These results are representative of three independent experiments.

Flow cytometry analysis of lung cells from wild-type C57BL/6 mice infected i.n. with 5x10^6^ CFU of wild-type or Δ*vir*B mCherry-*Br*, both stained with eFLuor, confirmed the presence of two well distinct populations of infected AMs at 48 hours post-infection (**Fig 4**). As AMs are auto-fluorescent at most wavelengths used in common fluorescence reporter systems, we chose to identify infected cells on the basis of the eFluor signal, which is particularly intense and easily detectable, as shown in **Fig 4A**. At 48 hours, >90% of eFluor^+^ cells appeared as CD11c^high^ Siglec-F^high^ AMs (**Fig 4B**). These cells can be divided into two distinct populations based on the mCherry-*Br* count measured by the intensity of the mCherry signal (**Fig 4C**). Kinetic analysis showed that the frequency of mCherry^high^ cells strongly increases between 12 and 48 hours post-infection (**Fig 4D**). At 48 hours post-infection, 10.6 ± 2.5% of eFluor^670+^ cells appeared to be mCherry^high^ permissive cells. Permissive and resistant cells were not distinguishable based on CD11b, CD11c, Ly6C, Ly6G, F4/80 and MHCII expression (**S1B Fig**). As expected, only a negligible frequency of mCherry^high^ cells were detected at 48 hours post-infection in the lungs of mice infected with the Δ*vir*B mCherry-*Br* strain (**Fig 4C** and **4D**).

On the whole, flow cytometry analysis of lung cells from infected mice demonstrated that growth of *Brucella* during pulmonary infection is observed only in a small fraction of infected alveolar macrophages. These permissive cells are thus thought to be responsible for establishing the infection and disseminating *B*. *melitensis* to the spleen.

At this stage of our study, we can therefore affirm that *B*. *melitensis*, despite a constant level of CFU in the lung of infected mice, is exposed to strong and complex selection pressures. The effect of the latter on *B*. *melitensis* is observable very early and allows the elimination of *B*. *melitensis* in the majority of infected alveolar macrophages. This model therefore seems to us to be optimal for identifying the bacterial genes allowing *B*. *melitensis* to escape the immune response.

### Identification by Tn-seq of essential *B*. *melitensis* genes in 2YT rich culture medium

The identification of genes essential for growth in a rich medium is a prerequisite to identification of the genes necessary for optimal intracellular *B*. *melitensis* multiplication.

As described previously [21] and detailed in the Materials and Methods, a *B*. *melitensis* 16M library of ~1.4x10^7^ random mini-Tn5 mutants was constructed. The library was grown on 2YT rich medium, colonies were collected and regrown on rich medium to discard genomic DNA of dead bacteria or bacteria not able to regrow. Note that subsequent infection steps also involve two rounds of culture on rich medium, one before infection to make the library and one after infection to recover the CFU. Transposon insertion sites were identified by Illumina sequencing. We identified 1,312,197 unique insertion sites from 778,858 (chromosome I) and 533,339 (chromosome II) mapped reads, saturating the *B*. *melitensis* genome with a unique insertion site every 2.5 bp on average.

In order to identify essential genes, both chromosomes were scanned with a sliding window of 100 bp, allowing the computing of a R100 score, corresponding to the log_10_ (number of aligned reads +1) (see Materials and Methods). Essential genes (ES) were those having at least one R100 value equal to 0. A list of all *B*. *melitensis* genes associated with the number of times the R100 value fell to 0 for the gene is shown in **S1 Table**. Of the 3,369 predicted genes annotated in the *B*. *melitensis* genome, 458 genes were found to be ES for *in vitro* culture in rich medium, i.e. 13.6% of the predicted genes.

The second *Brucella* chromosome has been postulated to originate from an ancestrally-acquired megaplasmid [24][26], and the distribution of ES genes in *B*. *abortus* is consistent with this hypothesis since the proportion of essential genes in chromosome I is higher compared to chromosome II [21]. Indeed, we found that 404 out of the 2,197 genes (18%) of chromosome I of *B*. *melitensis* were ES (**S1 Table**). This is 3.9 times more than the 4.6% found on chromosome II, with 54 ES genes out of 1,172 (**S1 Table**). This result further supports the megaplasmid hypothesis.

In order to perform a quantitative gene-by-gene analysis of 2YT rich medium condition, we also computed a transposon insertion frequency (**TnIF**) parameter, equal to the log_10_ (r+1) for a given coding sequence, with r = number of reads aligned in the central 80% of the coding sequence of the considered gene (see Materials and Methods). A list of all *B*. *melitensis* genes associated with a TnIF value for the 2YT rich medium condition (hereunder caller CTRL condition) is presented in **S1 Table**.

As explained in [27], the second mode of the frequency distribution of TnIF corresponds to the genes that are not affected in the CTRL condition. In the rest of our analyses, we only considered unaltered genes in 2YT CTRL condition, so only genes that are located in the second mode of the graphs showed in **S4 Fig**. Thus, only 2508 genes among the 3369 genes of *B*. *melitensis* will now be considered in our next Tn-seq analyzes in RAW 264.7 and mice.

In order to identify genes affected during an infection condition (cdt) compared to the CTRL condition, a ΔTnIF equal to TnIF-cdt–TnIF-CTRL value was calculated for each gene, where TnIF-cdt was the tested condition and TnIF-CTRL the 2YT rich medium control condition. The frequency distribution of ΔTnIF values was plotted for both chromosomes and for each condition tested in our study (**S5 Fig**).

### Differential requirement of genes for lung infection compared to the RAW 264.7 macrophage cell line *in vitro*

Wild-type C57BL/6 mice were intranasally infected with 5x10^6^ CFU of the 16M transposon mutant library described above and sacrificed at 5, 24, 48, 72 and 120 hours post-infection. RAW 264.7 macrophages were infected *in vitro* with the same library and harvested at 24 hours post-infection. At each time point of *in vivo* and *in vitro* infection, bacteria were extracted from the organ or cell culture and grown on 2YT rich medium. Bacterial colonies were collected, mixed, and used to extract genomic DNA on which a transposon insertion site identification was performed as indicated in the Materials and Methods.

The possible decrease of TnIF during the period of infection was scored gene by gene, and the TnIF obtained after two rounds of culture on rich medium was compared to the TnIF value obtained in a given infection condition, yielding a ΔTnIF value per gene and per condition. A list of all genes whose ΔTnIF < -0.5 in at least one condition tested is shown in **S2 Table**. In the following, we will refer to three categories of ΔTnIF values: unaffected (ΔTnIF>-0.5), **low fitness** (**LF**, -1.0< ΔTnIF<-0.5) and **very low fitness** (**VLF**, ΔTnIF<-1.0).

Since the course of *B*. *melitensis* infection in the lung was characterized over time by microscopy and flow cytometry (see above), we first compared different times post-infection to identify by Tn-seq the genes required at different stages of infection. We found that most genes that were LF or VLF at 120 hours in the lung were already LF or VLF at 48 hours (**S6A Fig**), which is consistent with CFU and microscopy analyses showing that *Brucella* faces intense selection pressures between 24 and 48 hours post-infection in the lung. This kinetic analysis indicates that no further strong gene-specific selection occurs between 48 and 120 hours post-infection, and that defects already present at 48 hours are only amplified at 120 hours post-infection. One notable exception are the genes involved in the synthesis of the core and the O chain of LPS. These genes were already predominantly LF at 5 hours post-infection in the lungs (**S6B Fig**), which suggests that the core and O chain are required very early in the lung to resist to host immune defenses. In comparison, *virB* genes became VLF only from 48 hours onward (**S6C Fig**).

**Fig 5:**
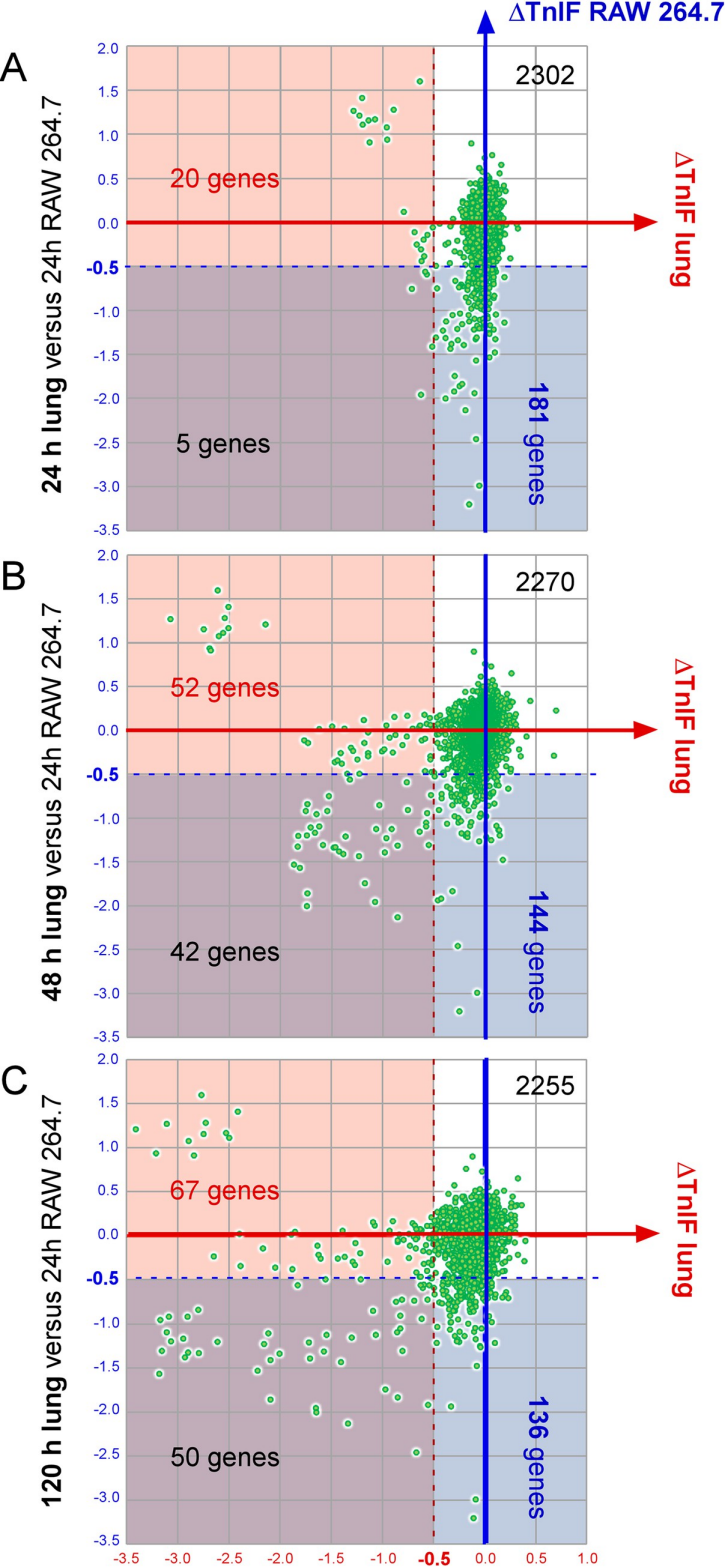
Comparison of *B*. *melitensis* genes required for optimal multiplication in the lung and RAW 264.7 macrophage conditions. The figure shows the distribution of the ΔTnIF values of all *B*. *melitensis* genes (n = 2508) which are predicted not to induce an attenuation of fitness in rich medium (CTRL). Each gene is defined by two ΔTnIF values. The x-axis indicates the ΔTnIF value of the lung (TnIF_lung_—TnIF_CTRL_) at 24 (**A**), 48 (**B**) and 120 (**C**) hours post infection. The y-axis indicates the ΔTnIF value in macrophage RAW 264.7 (TnIF_RAW 264.7_—TnIF_CTRL_) at 24 hours post infection. These ΔTnIF values comparisons allow to visualize all the genes associated with a drop in fitness both in the lung and macrophages (purple area), in the lung specifically (pink area) or in RAW 264.7 macrophages specifically (blue area). Numbers indicate the number of LF or VLF genes (ΔTnIF<-0.5) in each area.

We then compared the Tn-seq data generated at 24, 48 and 72 hours post-infection in the lung to those obtained at 24 hours post-infection in RAW 264.7 macrophages (**Fig 5A, 5B and 5C**). At 24 hours post-infection, 186 genes appear LF or VLF in RAW 264.7 macrophages (**Fig 5A**). This set of genes markedly differ from the set of 25 genes that appear necessary for *B*. *melitensis* survival at 24 hours post-infection in the lungs, since only 5 genes are common. There are therefore 181 genes LF or VLF specifically in RAW264.7 (blue area) and 20 genes LF or VLF specifically in lungs (red area). A strong difference is also observed at the other time points: 52 genes are LF or VLF specifically in lungs 48 hours post infection (**Fig 5B**) and 67 genes at 120 hours post infection (**Fig 5C**). This demonstrate that the differences between the two conditions are therefore not due to a difference in infection kinetics, but reflects the very different selection pressures encountered by *B*. *melitensis* in lung and RAW 264.7 macrophages.

**Fig 6:**
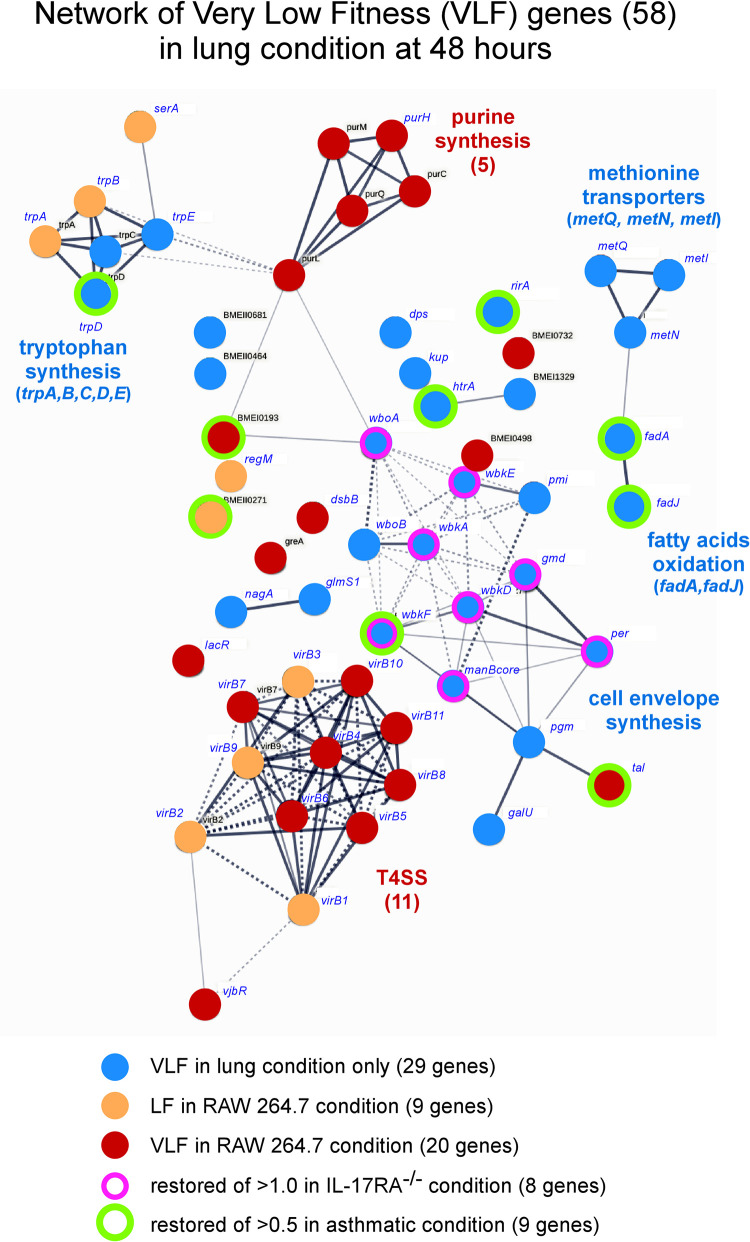
Clustering analysis of very low fitness genes in the lung condition at 48 hours post-infection. The diagram shows the potential interactions between the 58 genes displaying a ΔTnIF < -1.0 identified in lungs of wild-type mice at 48 hours post-infection. The color code of the legend indicates whether the genes are specifically attenuated in the lung or if they are also attenuated in the RAW 264.7 cell line condition at 24 hours post-infection. The legend also indicates which genes are predicted to be partially restored in IL-17RA^-/-^ mice and asthmatic mice conditions. This clustering analysis was carried out with STRING (https://string-db.org).

**Fig 7:**
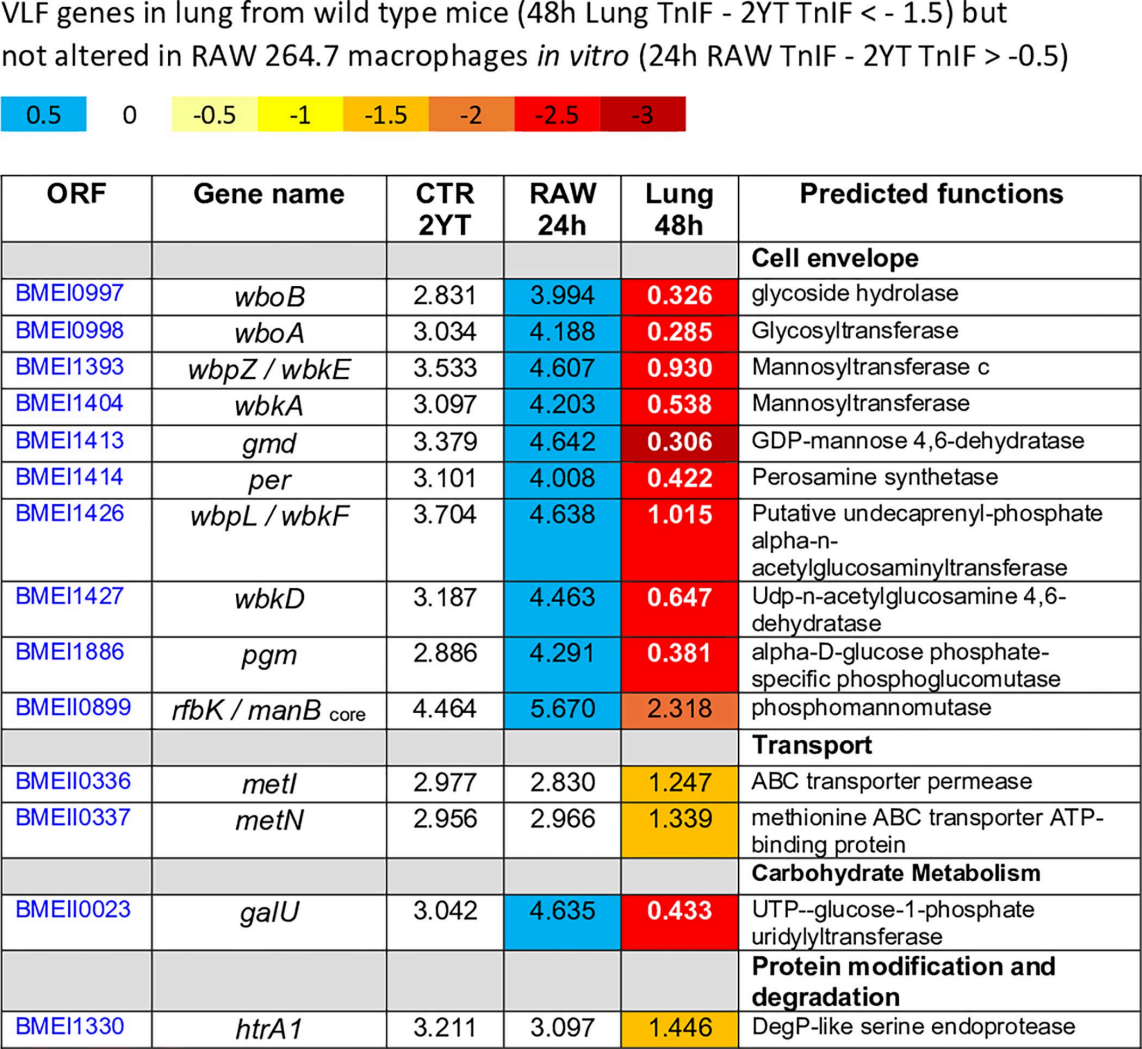
List of 14 Very Low Fitness (VLF) genes specific to the lung condition (ΔTnIF lung 48 hours < -1.5, ΔTnIF RAW 264.7 > -0.5) with TnIF values in the 2YT, RAW 264.7 and lung conditions, as well as a predicted function. Color scale shows the score of ΔTnIF of each gene.

As a large number of daughter cells is observed only from 48 hours post-infection in the AMs (**Fig 2B**), we choose to focus our comparison between 24 hours RAW 264.7 and 48 hours lung conditions. At 48 hours, a total of 36 LF and 58 VLF genes were predicted in lung condition. Among these 58 VLF genes, 29 are specific to lung and 29 are also LF or VLF in RAW 264.7 condition (**Fig 6**). Among the 29 VLF genes specific of lung, 14 genes display a very strong drop of their TnIF value (TnIF < -1.5) and are listed in **Fig 7**. These genes include 10 genes involved in LPS core and O chain synthesis, two genes, *metI* and *metN*, predicted to code for methionine transporters, *galU* (UTP—glucose-1-phosphate uridylyltransferase) that catalyzes the synthesis of UDP-glucose, an essential metabolite playing a key role in the synthesis of the components of the bacterial envelope, particularly the lipopolysaccharide and the capsule [28] and *htrA1* which is a stress response serine protease implicated in the resistance to oxidative damage [29]. In striking contrast, all VLF genes implicated in LPS synthesis, as well as *galU*, were predicted by Tn-seq to display a hyperinvasivity phenotype in RAW 264.7 macrophages, as previously reported for *B*. *abortus* [21] and in agreement with cellular infections previously performed with *Brucella* rough mutants [30][31], further demonstrating the strong difference between lung and RAW 264.7 macrophages infection models.

To validate our Tn-seq predictions, we constructed several untagged deletion mutants. We chose to construct mutants for *gmd* (BMEI1426), *per* (BMEI1414) and *wbkF* (BMEI1426) that are implicated in LPS O chain biosynthesis and are predicted to be VLF in the lung but hyperinvasive in RAW 264.7 macrophages. We also constructed mutants for *fadA* and *fadJ* (BMEII0496 and BMEII0497) and *trpD* (BMEI0844) genes involved in fatty acid oxidation and tryptophan synthesis, respectively. These three genes are predicted to be specifically VLF in the lung. As negative control, we used a mutant of the complete *virB* operon that is strongly attenuated in both infection models. As positive controls, we used the wild-type *B*. *melitensis* strain and a *glpX* (BMEI0726) mutant that is predicted to be not significantly attenuated in both conditions according to our Tn-seq analyses (**S1** and **S2 Tables**).

**Fig 8:**
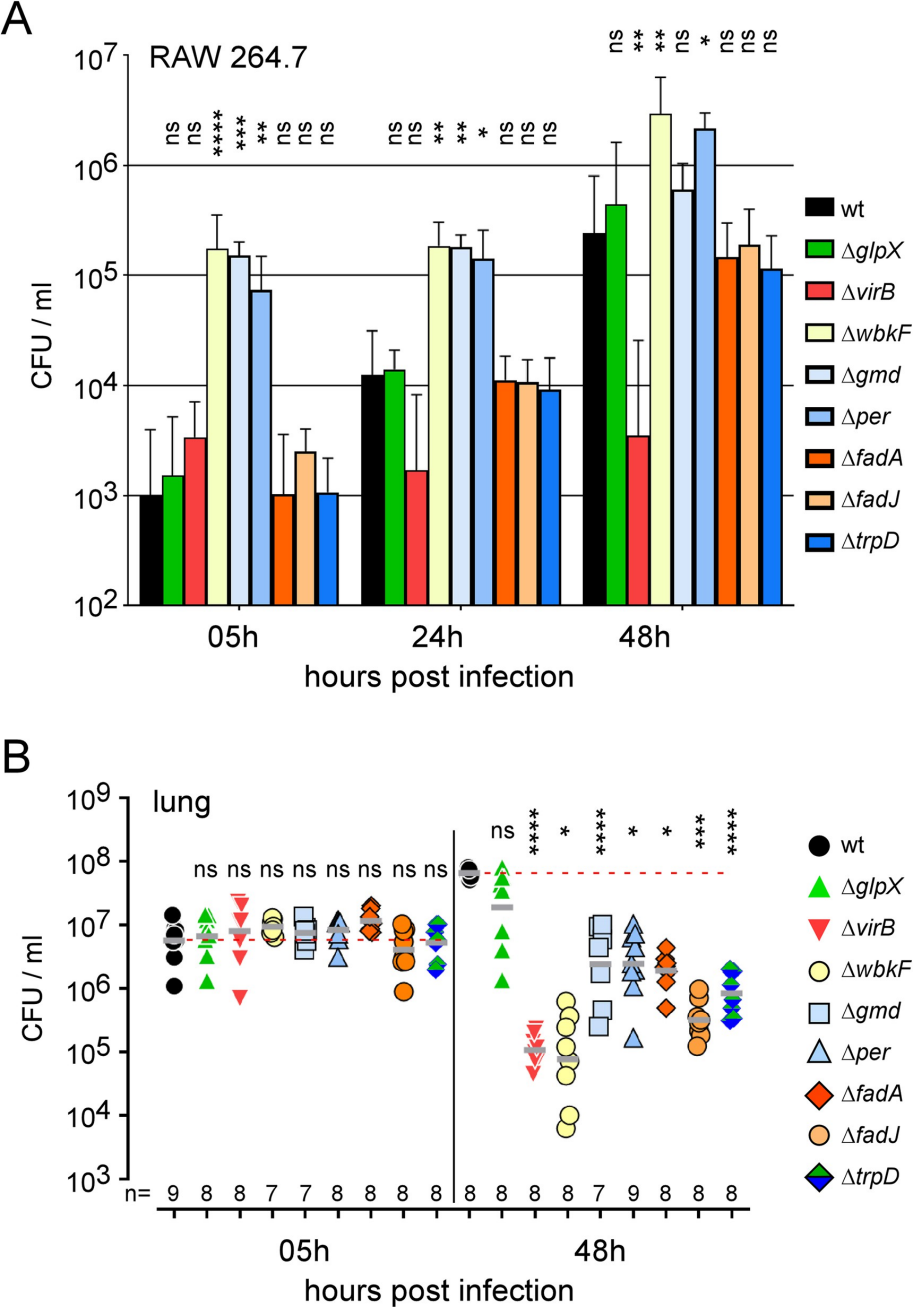
Functional confirmation of the prediction derived from the Tn-seq analysis of RAW 264.7 and lung conditions. Data shown are bacterial count (CFU) at the indicated time post-infection in RAW 264.7 (**A**) and lung from wild-type mice infected intranasally (**B**) with wild-type (wt), Δ*glpX*, Δ*virB*, Δ*wbkF*, Δ*gmd*, Δ*per*, Δ*fadA*, Δ*fadJ* and Δ*trpD* strains of *B*. *melitensis* at a dose of 5x10^6^ CFU and a MOI of 1:50, respectively. Significant differences between wt and the indicated groups are marked with asterisks: *p < 0.1, **p < 0.01, ***p < 0.001, ****p < 0.0001, in a One-Way ANOVA with Kruskal-Wallis post-test. These results are representative of three independent experiments.

In RAW 264.7 macrophages, Δ*gmd*, Δ*per* and Δ*wbkF* mutants appeared hyperinvasive although the Δ*virB* mutant was significantly attenuated and the Δ*glpX* mutant did not differ significantly from the wild-type strain (**Fig 8A**). In contrast, all mutants appeared significantly and strongly attenuated in the lung at 48 hours post-infection compared to the wild-type strain, with the exception of the Δ*glpX* mutant which showed very low attenuation, as expected (**Fig 8B**).

Collectively, the Tn-seq data demonstrate that different sets of bacterial genes are needed to survive to intracellular selective pressure encountered in the RAW 264.7 macrophages infection model and in the mice infection model.

### Host immune status affect the nature of essential bacterial genes

**Fig 9:**
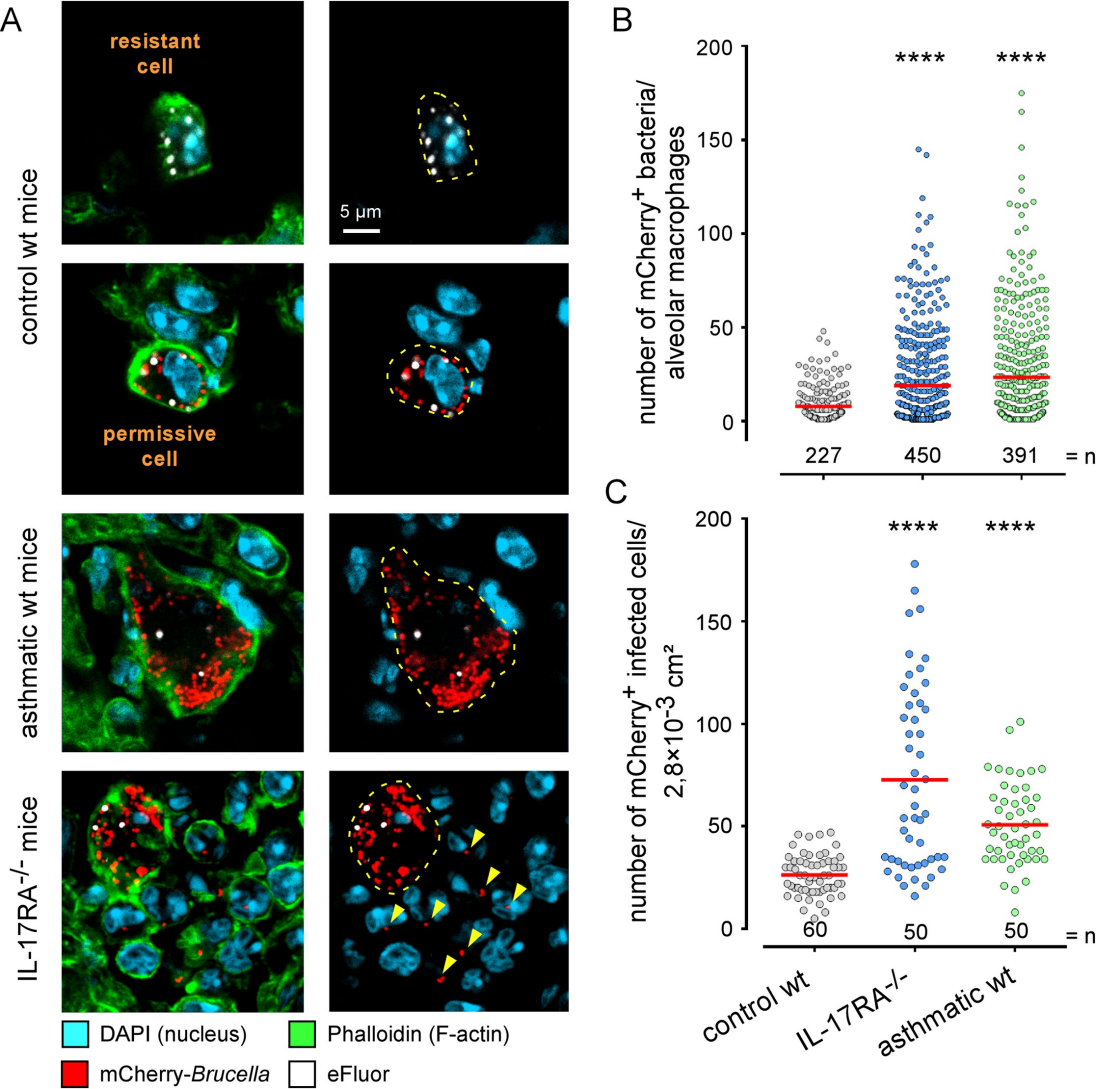
IL-17RA deficiency and asthma induce distinct patterns of infection in the lung. Control and asthmatic wild-type C57BL/6 mice and IL-17RA^-/-^ mice were infected intranasally with a dose of 5x10^7^ CFU of mCherry-*B*. *melitensis* labelled with eFluor^670^. Mice were sacrificed at 48 hours post-infection and the lungs were collected and analyzed by confocal and fluorescent microscopy for the expression of DAPI, phalloidin, mCherry and eFluor^670^ markers. Data shown are (**A**) representative confocal images (single z-plane) of infected cells, (**B**) the number of mCherry^+^ bacteria / alveolar macrophages and (**C**) the number of mCherry^+^ bacteria per lung surface unit determined by fluorescent microscopy. In order to avoid biases in the analysis, an automatic acquisition of the entire surface of the tissue section is carried out using MosaiX module from AxioVision program (Zeiss). We exclude from this analysis the edges of the organ as well as the damaged areas. n indicates the number of cells analyzed (**B**) or the number of surfaces analyzed (**C**) for each condition. Significant differences between control wt and the indicated groups are marked with asterisks: ****p < 0.0001, in a One-Way ANOVA with Kruskal-Wallis post-test. The data are representative of two independent experiments.

We hypothesize that some of the additional selection pressures that *B*. *melitensis* experiences during the first 48 hours of infection in the lung are due to the immune system. In order to determine the relative importance of the different types (i.e. T helper (Th)1, Th2, Th17) of immune response, we compared the course of *B*. *melitensis* in wild-type C57BL/6 and IFNγR^-/-^, TNFR1α^-/-^, IL-4^-/-^, IL-17RA^-/-^ and asthmatic wild-type mice (**S7A Fig**). Comparison of the CFU counts at 48 hours post-infection showed that IL-17RA^-/-^ mice and asthmatic mice, but not TNFR1α^-/-^, IL-4^-/-^ and IFNγR^-/-^ mice, lose control of early *B*. *melitensis* infection. Using mCherry-*Br* stained with eFluor, we confirmed by flow cytometry (**S7B Fig**) and confocal microscopy (**Fig 9**) that the higher susceptibility of IL-17RA^-/-^ mice and asthmatic mice was correlated to a higher frequency of mCherry^high^ AMs at 48 hours post-infection, compared to wild-type mice. Interestingly, confocal analysis (**Fig 9A**) showed that IL-17RA deficiency leads not only to an increase of the frequency of highly-infected cells but also to higher dissemination of bacteria in the lung. Indeed, a higher frequency of weakly-infected cells is usually observed in IL-17RA^-/-^ mice near highly-infected cells. In contrast, asthma mainly induces very large hyper-infected cells (**Fig 9A**). These differences were highlighted by counting the number of bacteria per cell (**Fig 9B**) and the number of infected cells per unit area (**Fig 9C**). IL-17RA deficiency was associated with a moderate increase of bacteria per cell and a strong increase of the number of infected cells.

To identify VLF genes specifically involved in resistance to the immune response, we chose to perform a Tn-seq analysis on lungs from IL-17RA^-/-^ and asthmatic mice infected for 48 hours. Since the phenotype of infected cells in these susceptible mice is different, as described above, they are unlikely to generate identical selection pressures for *B*. *melitensis*. Wild-type, IL-17RA^-/-^ and asthmatic C57BL/6 mice were intranasally infected with 5x10^6^ CFU of our *B*. *melitensis* 16M mini-Tn5 mutant library and sacrificed at 48 hours post-infection. A list of all genes presenting a significant drop of the TnIF value for each condition tested is shown in **S2 Table**.

**Fig 10:**
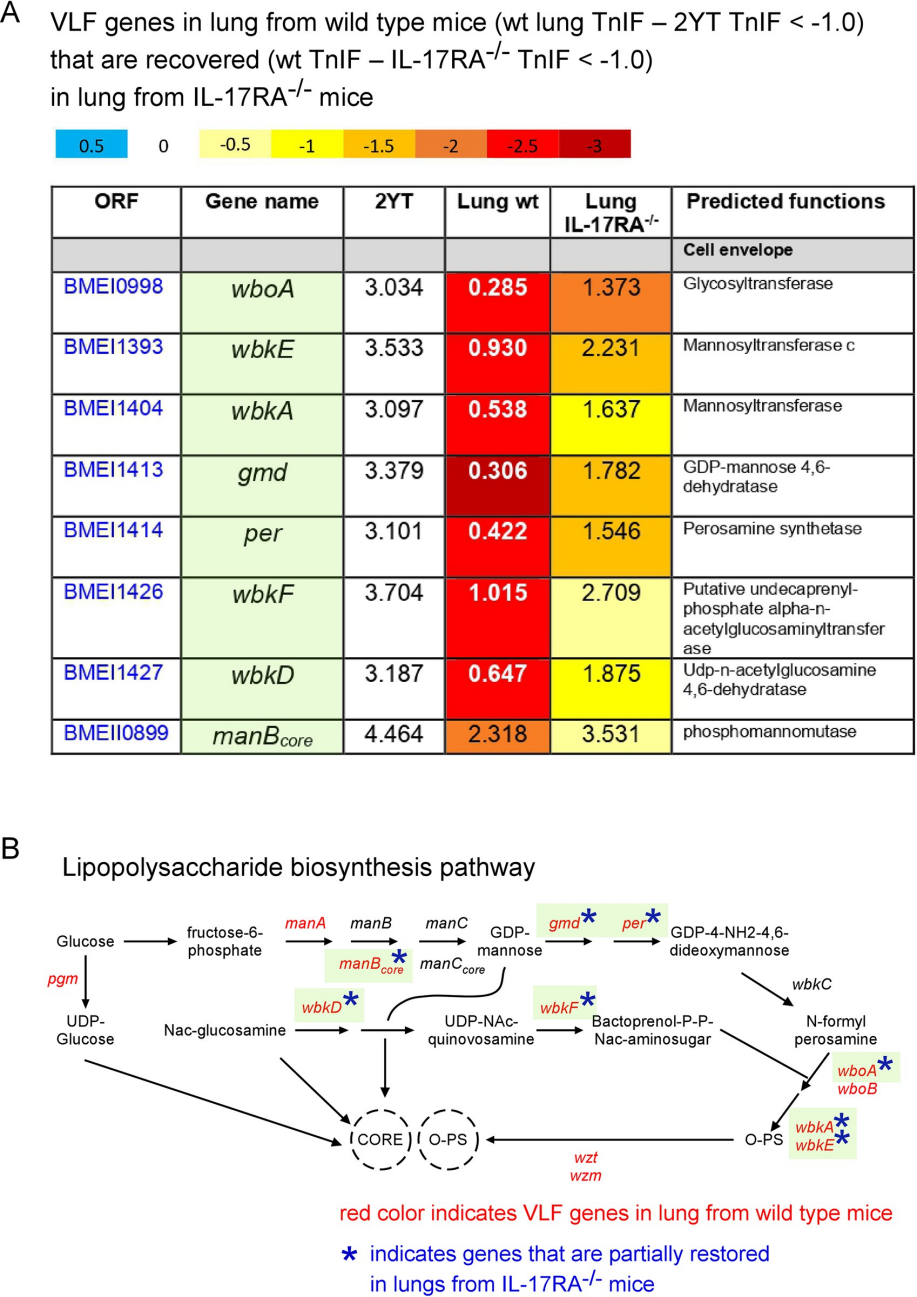
Comparison of *B*. *melitensis* genes required for optimal multiplication in lungs of wild-type and IL-17RA^-/-^ mice. **A**. List of VLF genes in lungs of wild-type mice (< -1.0 ΔTnIF) that are recovered (> 1.0) in lungs of IL-17RA^-/-^ mice associated with their TnIF values in the CTRL 2YT and lung from wild type and IL-17RA^-/-^ mice conditions, as well as predicted function. **B**. Schematic representation of the lipopolysaccharide biosynthesis pathway of *Brucella* specifying the genes that become partially dispensable in the IL-17RA^-/-^ mice condition (adapted from the KEGG PATHWAY database, https://www.genome.jp/kegg/pathway.html).

We found 8 VLF genes for wild-type lung conditions displaying an increase >1.0 of their TnIF in the IL-17RA^-/-^ lung condition (**Figs 6 and 10A**), meaning that these mutants have a better chance to survive or multiply (or both) upon infection of IL-17RA^-/-^ mice. These 8 genes are implicated in LPS O chain biosynthesis (**Fig 10B**).

We also found 9 LF or VLF genes for wild-type lung conditions displaying an increase >0.5 of their TnIF in the asthmatic lung condition (**Fig 6**). These genes belong to many functional categories. Among them, we found genes implicated in tryptophan synthesis (*trpD*), LPS synthesis (*wbkF*) and in β-oxidation of fatty acids (*fadA*, *fadJ*). The differential requirement of tryptophan and fatty acid metabolism genes suggests a different nutritional context between the wild-type and asthmatic conditions.

**Fig 11:**
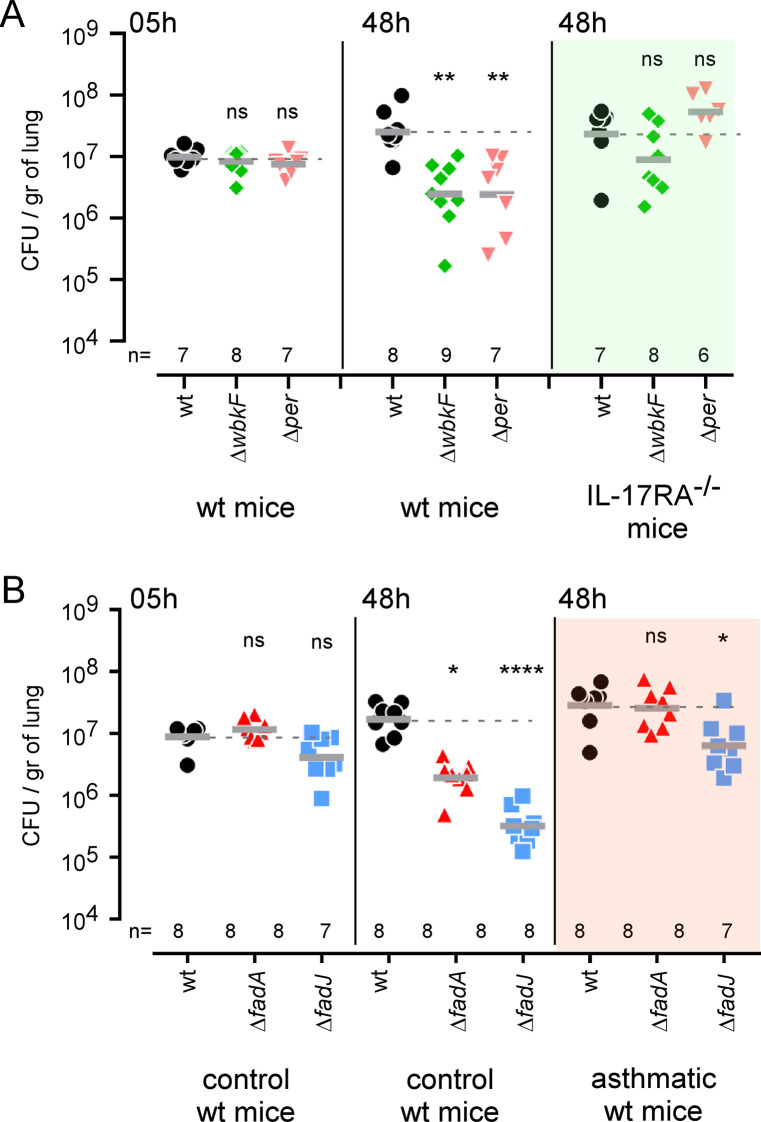
Functional confirmation of the prediction derived from the Tn-seq analysis of the IL-17RA^-/-^ and asthmatic conditions. **A.** Data shown are bacterial count (CFU) at 5 and 48 hours post-infection in wild-type and IL-17RA^-/-^ mice infected intranasally with wild-type (wt), Δ*wbkF* and Δ*per* strains of *B*. *melitensis* at a dose of 5x10^6^ CFU. **B.** Data shown are bacterial count (CFU) at 5 and 48 hours post-infection in control and asthmatic wild-type mice intranasally infected with wild-type, Δ*fadA* and Δ*fadJ* strains of *B*. *melitensis* at a dose of 5x10^6^ CFU. Significant differences between wt and the indicated groups are marked with asterisks: *p < 0.1, **p < 0.01, ****p < 0.0001, in a One-Way ANOVA with Kruskal-Wallis post-test. These results are representative of three independent experiments.

To test our Tn-seq predictions, we compared the course of Δ*wbkF* and Δ*per* (LPS O chain biosynthesis) and Δ*fadA* and Δ*fadJ* (β-oxidation of fatty acids) with a wild-type *B*. *melitensis* strain in wild-type, IL-17RA^-/-^ and asthmatic mice, by counting the CFU 5 and 48 hours post-infection. We observed partial restoration of Δ*wbkF* and Δ*per* in IL-17RA^-/-^ mice (**Fig 11A**) and of Δ*fadA* and Δ*fadJ* in asthmatic mice (**Fig 11B**), as expected from the Tn-seq data.

## Discussion

Understanding how pathogens adapt to the *in vivo* environment is a fundamental question in biology that also has important implications for the treatment of diseases and the selection of vaccine candidates. This work describes the first use of a saturating transpositional library of *B*. *melitensis* 16M to identify genes required during the early phase of *B*. *melitensis* infection in a mouse model. In order to discriminate between core genes and genes required in specific conditions of infection, we analyzed and compared a total of 8 different conditions including *B*. *melitensis* growth in 2YT rich medium, RAW 264.7 macrophages and the lungs of infected wild-type and genetically-deficient C57BL/6 mice.

Our objective in this work was not to carry out a formal comparison between the *in vitro* and *in vivo* conditions. RAW 264.7 macrophages are derived from BALB/c mice and are presumably very different from alveolar macrophages found in the lungs from C57BL/6 mice since C57BL/6 mice are known to be more resistant to *B*. *melitensis* infection than BALB/c mice [32]. We have chosen to use RAW 264.7 only because it is a classical model for the study of *Brucella* infection *in vitro*. We have previously shown in the mouse infection model that the control of *B*. *melitensis* infection in the lung involves immune mechanisms distinct from those of the spleen or the footpad [10]. It should therefore be obvious that the lungs represent a specific compartment of the body which cannot be considered representative of the whole of it. We chose to analyze the lung because it represents a natural entry route for *B*. *melitensis* and because in the lungs *B*. *melitensis* multiplies mainly in the alveolar macrophages whereas in the spleen and the footpad *B*. *melitensis* multiplies in multiple cell types [10], making it more difficult to monitor cellular infection by transmission electron microscopy. Thus, our objective was to compare two different reference experimental models, each having its own limitations and being probably far removed from the conditions encountered by *B*. *melitensis* in its natural hosts.

First, we identified 861 genes among 3369 that are predicted to be required for optimal growth of *B*. *melitensis* in 2YT rich medium in the absence of any selection pressure. We then tried to identify the genes required for survival of *B*. *melitensis* in phagocytic cells *in vitro*. We identified 186 additional LF or VLF genes necessary for optimal multiplication of *B*. *melitensis* in RAW 264.7 macrophages reference model. Our results mainly confirm the results obtained previously by Tn-seq analysis of *B*. *abortus* multiplication in rich media and in RAW 264.7 macrophages [21].

Our detailed comparative microscopic analysis of the multiplication of *B*. *melitensis* in RAW 264.7 macrophages and in the lung of intranasally infected mice demonstrates that *B*. *melitensis* encounters much more intense and complex selection pressures in the lung than in RAW 264.7 macrophages. We discovered that in the lung, *B*. *melitensis* seems to undergo a phase of intense selection after an initial growth phase inside AMs. While growing bacteria are detected by fluorescent microscopy at 12 hours post-infection, dead bacteria are already observable in large amounts in AMs at 24 hours post-infection, which is not the case in RAW 264.7 macrophages. Surprisingly, growing bacteria and daughter bacteria are selectively eliminated in alveolar macrophages since only non-growing bacteria are still alive at 24 h PI. Although killing mechanisms involved at this stage are not known, it is likely that the variety of behaviors of *B*. *melitensis*, growing or remaining in a dormant state, can constitute a selective advantage for the bacterium. Bacteria growing too early being eliminated and bacteria growing too late being potentially lost in the competition between bacteria. Moreover, transmission electron microscopy showed that bacteria inside the alveolar macrophages have already an altered morphology after a few hours of infection, that we correlate to a state of stress. We observed that *B*. *melitensis* cultured in Plommet medium grow at a much slower rate than in rich 2YT medium, suggesting that they are under nutritional stress. *B*. *melitensis* cultured in Plommet present in TEM a particular morphology that we also observe within AMs, suggesting that some *B*. *melitensis* encounter nutritional stress in these cells. We also observed in AMs an atypical bacterial morphology, which we named "dark". These later present a very dense cytoplasm, refractory to electrons emitted by the TEM. These forms are not observed in RAW 264.7 in our experimental conditions. They could correspond to an adaptation of *B*. *melitensis* to certain stresses encountered in AMs. The high density of the cytoplasm could result from the association of bacterial DNA with stress proteins in order to protect it from damage. It has been shown that the Dps protein from *Escherichia coli*, which is highly conserved in bacteria, can non-specifically associate with bacterial DNA and form crystal micro assemblies [33]. This association would be reversible and would not require the synthesis of new proteins [34]. Several pathogenic intracellular bacteria express proteins similar to Dps. The Dps-like protein *Fri* from *Listeria monocytogenes* has been associated with resistance to intracellular stresses [35]. *Mycobacterium smegmatis* expresses a Dps-like protein under carbon starvation conditions which associates with its DNA to form a crystal structure [36]. The Dps-like protein of *Brucella microti* is required for survival in acidic conditions [37]. We could therefore propose the hypothesis that, in response to specific stresses encountered in AMs, certain *B*. *melitensis* adopt a "dark" morphology allowing them to better resist stresses affecting DNA. Accordingly, our Tn-seq analysis of *B*. *melitensis* (**S2 Table**) predicts that deficiency of Dps protein (BMEI1980) would lead to attenuation in lungs at 48 hours post infection but not in RAW 264.7 macrophages. It would therefore be interesting to test whether a Dps deficiency could actually affect the multiplication of *B*. *melitensis* in AMs and reduce the frequency of bacteria with a "dark" morphology.

The complex pattern of bacterial killing, stress and growth observed *in vivo* shows that in the first 48 hours of infection, *B*. *melitensis* undergoes *in vivo* selection pressures that are absent *in vitro*, at least with the RAW 264.7 macrophages. Consequently, we performed a Tn-seq analysis at 24 hours post infection on RAW 264.7 and at 5, 24, 48, 72 and 120 hours post-infection on lung from infected mice. Many of the genes predicted as LF or VLF in the lung were not predicted to be attenuated in RAW 264.7 macrophages. For example, among the 94 genes identified by Tn-seq as LF or VLF in the lungs of mice infected for 48 hours, 52 are specific to the lung condition and are not predicted by Tn-seq as attenuated in RAW 264.7 macrophages. Among the 20 genes displaying the higher decrease of TnIF in lung condition but not in RAW 264.7 condition, we found genes implicated in tryptophan synthesis (*trpD*), LPS synthesis (*gmd*, *wboA*, *wbkF*, *per*, *wbkE*, *wbkA*, *wbkD*, *pgm*, *wboB*, *galU*, *manB*_*core*_), methionine transport (*metI*, *metN*), potassium transport (*kup*) and *htrA1*, a serine protease. We also found genes implicated in carbohydrate (*glmS1*, *pmi*) and fatty acid (*fadA*, *fadJ*) metabolism, suggesting that *B*. *melitensis* seems to encounter specific nutritional conditions in the lung. Among these genes, Δ*gmd*, Δ*wboA*, Δ*wbkF*, Δ*per*, Δ*wbkE*, Δ*wbkA*, Δ*wbkD*, Δ*pgm*, Δ*wboB*, Δ*manB*_*core*_ [38] and Δ*htrA1* [39] strains have been described as attenuated in the spleen of BALB/c mice following intraperitoneal infection. To our knowledge, the impact of the deficiency of the *trpD*, *metI*, *metN*, *kup*, *galU*, *glmS1*, *pmi*, *fadA*, *fadJ* genes on *Brucella* virulence in mice has not been described. In our intranasal infection model in C57BL/6 mice, we confirmed the attenuation of *B*. *melitensis* Δ*wbkF*, Δ*gmd*, Δ*per*, Δ*fadA*, Δ*fadJ and* Δ*trpD* mutants in the lungs at 48 hours post-infection and the absence of their attenuation in RAW 264.7 macrophages. Thus, the available bibliographic data as well as our mutant tests largely confirm both the predictions of our Tn-seq analyses and their novelty.

In order to better understand the nature of the selection pressures encountered by *B*. *melitensis* in the lung, we compared the multiplication of *Brucella* in mice genetically deficient for key elements of the immune response. We confirm in our experimental model previous observations [9] showing that early infection control in the lung is dependent on IL-17RA but not of IFNγR. Interestingly, some genes implicated in LPS synthesis, such as *wbkF* and *per*, which could also be involved in the biosynthesis of native hapten [40], are indispensable *in vivo* at 48 hours post-infection in wild-type mice but not in IL-17RA^-/-^ mice. This suggests that the bacterial envelope is critical to resistance to the early anti-bacterial IL-17RA-dependent immune response, such as antimicrobial peptides [41].

We have shown previously [11] that *B*. *melitensis* multiply more intensively in the lungs of asthmatic C57BL/6 mice. This increased susceptibility requires a functional IL-4/STAT-6 signaling pathway. In order to determine whether the Th2 biased immune status of a wild-type mouse can alter the nature of the genes essential to optimal multiplication, we compared the bacterial genes required in control and asthmatic C57BL/6 mice. We found 9 genes, belonging to many functional categories, that display an increase >0.5 of their TnIF in the asthmatic lung condition when compared to control lung condition. Interestingly, among them several genes are implicated in tryptophan biosynthesis and the β-oxidation of fatty acids, suggesting that tryptophan and products of fatty acid degradation become available for *Brucella* in infected cells in the asthmatic condition. IL-4/STAT6 signaling is known to induce peroxisome proliferator-activated receptor γ (PPARγ) expression that leads to increased oxidation metabolism of fatty acids in macrophages (reviewed in [42]) and *B*. *abortus* has been demonstrated to survive and replicate preferentially in bone marrow-derived macrophages that were treated with IL-4 to differentiate in alternatively activated macrophages [43]. In this model, PPARγ activity was identified as a causal mechanism promoting enhanced *Brucella* survival.

On the whole, our first functional mapping of the genome of *B*. *melitensis in vivo* validates the effectiveness of the Tn-seq approach in establishing a link between the genotype and phenotype. We demonstrate that the immune status of the infected animal determines which genes are required for optimal growth. Many of the genes identified are involved in bacterial metabolism, suggesting that both the immune response and the nature of the infected cell type determine the available nutrients for *Brucella*.

## Materials and methods

### Ethics statement

The procedures used in this study and the handling of the mice complied with current European legislation (Directive 86/609/EEC). The Animal Welfare Committee of the Université de Namur (UNamur, Belgium) reviewed and approved the complete protocol for *Brucella melitensis* infection (Permit Number: UN-LE-18/309).

### Mice and bacterial strains

Wild-type C57BL/6 mice were acquired from Harlan (Bicester, UK). IFN-γR^-/-^ C57BL/6 mice[44] were acquired from Dr B. Ryffel (University of Orleans, France). IL17RA^-/-^ C57BL/6 mice [45] were acquired from Dr O. Denis (Belgian Scientific Institute for Public Health, Brussels, Belgium). TNFR1^-/-^ C57BL/6 [46] were acquired from Dr C. De Trez (Vrije Universiteit Brussel). IL4^-/-^ C57BL/6 mice were purchased from The Jackson Laboratory (Bar Harbor, ME). All wild-type and deficient mice used in this study were bred in the animal facility of the Gosselies campus of the Université Libre de Bruxelles (ULB, Belgium). The wild-type *B*. *melitensis* 16M strain used here is a Nal^R^ derivative of wild-type *B*. *melitensis* 16M [47]. We also used *B*. *melitensis* 16M stably expressing a rapidly maturing variant of the red fluorescent protein DsRed [48], the mCherry protein, under the control of the strong *Brucella* spp. promoter p_*secE*_, also called p_*sojA*_. The construction of the mCherry-producing *B*. *melitensis* (mCherry-*B*) strain has been described previously in detail [49]. The Δ*virB* mutant was constructed in the mCherry-*B* strain by triparental mating to introduce the pJQ200 UC1-*virB* plasmid from the *E*. *coli* DH10B strain (described in [23]) into the mCherry-*B* strain using the *E*. *coli* MT 607 (*pro-82 thi*-I *hsdR17* (r-m+) *supE44 recA56* pRK600) strain (described in [50]), and the allelic replacement was performed as described previously for other gene deletions [51]. Deletion of the *virB* operon was checked by PCR using the *virB*-F-check 5’-CGCTCGGCTATTATGACGGC-3’ and *virB*-R-check 5’-CGCCGATCATAACGACAACGG-3’ primers.

Cultures were grown overnight with shaking at 37°C in 2YT liquid medium (LB 32 g/L Invitrogen, Yeast Extract 5g/L, BD and Peptone 6 g/L, BD) and were washed twice in RPMI 1640 (Gibco Laboratories) (3500g, 10 min) before inoculation of the mice.

*Brucella melitensis* was always handled in BSL-3 containment according to Council Directive 98/81/EC of 26 October 1998 and the law of the Walloon government of 4 July 2002.

### Bioscreen analysis

Overnight cultures were prepared in 2YT rich medium the day before in order to obtain an OD_600 nm_ between 0.2–0.5 the day after. Cultures were washed twice in PBS (2000 g for 10 min at RT), and then suspended in 2 YT rich medium or Plommet-Erythritol minimal medium [52] to obtain an OD_600 nm_ of 0.05 in a final volume of 700 μL. We used the Bioscreen system (Thermo Fisher) to measure the growth of *Brucella* at 37°C for 72 hours.

### Allergens and allergic asthma sensitization protocol

*A*. *alternata* (strain 18586) (abbreviated Alt) was obtained from the BCCM/IHEM (Institute of Public Health, WIV-ISP, Brussels, Belgium) and cultured for 3 weeks at 27°C in flasks containing 250 ml of Czapek medium. Mold pellicles were harvested and homogenized in 0.4% NH_4_HCO_3_ + polyvinyl polypyrrolidone (Sigma) with an Ultra-Turrax. The homogenates were then shaken for 3 hours at 4°C. Extracts were centrifuged twice for 30 min at 20,000 g, dialyzed against phosphate-buffered saline (PBS) and stored at −20°C in 50% glycerol.

For asthma sensitization, mice were lightly anesthetized with isoflurane [from Abbott laboratories (# No. B506)]. Once the mice were unresponsive but breathing comfortably, a solution of Alt (5 μg of *A*. *alternata* extract in 100 μl of PBS) was applied directly to the nostrils. The animals were allowed to slowly inhale the liquid and then to recover in a supine position. Mice received the extract twice per week throughout the experiment, as described previously [11]. Mice were infected 17 days after the first instillation.

### Transposon mutagenesis

One milliliter of an overnight culture of a nalidixic acid-resistant strain of *B*. *melitensis* 16M was mixed with 50 μl of an overnight culture of the conjugative *Escherichia coli* S17-1 strain carrying the pXMCS-2 mini-Tn*5* Kan^r^ plasmid [21]. This plasmid possesses a hyperactive Tn*5* transposase allowing the straightforward generation of a high number of Tn mutants, as described previously [21]. The mating mixture was incubated overnight at room temperature (RT) on 2YT agar plates (rich medium, 1% yeast extract, 1.6% peptone, 0.5% NaCl, 2% agar). The resulting *B*. *melitensis* Tn mutants were selected on 2YT agar plates supplemented with both kanamycin (10 μg/ml) and nalidixic acid (25 μg/ml). Tn*5* mutagenesis generates insertion of the transposon at only one locus per genome, as demonstrated previously for *Brucella* [16].

### Analysis of essential genes for growth

Genomic DNA was extracted from each transposon library using standard techniques and prepared for the transposon library sequencing. Briefly, *B*. *melitensis* Tn mutants from each plate were collected, mixed and killed by heat (1 h, 80°C). The lysate was incubated 3 days at 37°C with a mix composed of Tris (tris-hydroxymethyl-aminomethane 50 mM), EDTA (Ethylenediaminetetraacetic acid, 50 mM), 0.1 M NaCl, Proteinase K (20 mg/mL) and 10% SDS. The mixture was treated with an equal volume of isopropanol 100% to precipitate the DNA, which was washed with ethanol 70%. Genomic DNA was resuspended in deionized water and genomic DNA flanking the Tn*5* was sequenced (Fasteris company, Geneva, Switzerland). In order to map mini-Tn*5* insertion sites, libraries were sequenced on an Illumina HiSeq with a primer hybridized at the border of the transposon, with its 3’ end pointing toward the flanking genomic DNA. The control Tn-seq on 2YT plates was repeated with an independent mini-Tn*5* library, with the same approach but with an Illumina NextSeq sequencing, generating very similar data (**S4C Fig**). Raw reads were mapped on *B*. *melitensis 16M* (accession numbers NC_003317 and NC_003318 for chromosomes I and II, respectively) using BWA [53] and read counts were determined using the samtools suite [54]. Libraries were read-depth normalized. To account for truncated but functional products and misannotated start sites, only insertions in the central 80% of each gene were considered. To determine essential genes, we computed a parameter called R100, defined as log_10_(number of Tn*5* insertions + 1) for a 100-nucleotides (nt) sliding window. This sliding window was shifted every 5 nt to generate a collection of R100 values spanning the whole genome for the control condition, i.e. bacteria on plates. Given that the *B*. *melitensis* genome is 3,281,397 base pairs (bp), a list of 656,397 R100 values was created, with an average of 2,511 transposon insertions mapped per window of 100 bp. As published previously [55], the probability of obtaining a window of a given size with no transposon insertion event can be estimated by the formula *P* = [1 - (w/g)]^n^ where w is the window size, g is the genome size, and n is the number of independent Tn*5* insertion events. In our case, the resulting probability was 3.8 x 10^−15^, with g = 3,281,397, w = 100, and n = 646,945. It should be noted that this value accounts only for a single window, whereas essential genes are typically characterized by a series of overlapping empty windows rather than a single 100-bp window, thus further lowering the probability of finding such profiles fortuitously. Essential genes were defined as all genes having at least one R100 value equal to 0. Defined essential genes usually have many R100 values equal to 0. Proven essential genes *ctrA* [56], *chpT* [57], *ccrM* [58], *divK* [59], and *omp2b* [60] are found as essential in this Tn-seq analysis, validating the detection of essential genes.

Mice were anesthetized with a cocktail of Xylazine (9 mg/kg) and Ketamine (36 mg/kg) in PBS before being inoculated intranasally with *B*. *melitensis* in 30 μl of RPMI. Tn-seq analysis of the mice intranasal model was performed by infecting 30 mice with a dose of 5x10^6^ CFU each, thus a total of 1.5x10^8^ clones, in order to cover the complexity of the libraries that were independently generated for each time post-infection. At each time post-infection, lungs were recovered, homogenized, and lysates were plated on 2YT kanamycin agar plates. Colonies were pooled, and mini-Tn5 insertion sites were mapped the same way as the 2YT control condition.

To determine if an insertional mutant in a defined gene is affected in a condition but untouched in the control 2YT condition; each gene was assigned an insertion index, called the **transposon insertion frequency** (**TnIF**), equal to the log_10_ of its total read count (thus including multiple insertions at the same position) divided by its length (in bp), corresponding on the 80% of the internal segment of the coding sequence. For each gene, a ΔTnIF (TnIF_cdt_−TnIF_CTRL_) value was calculated, where TnIF was computed for the tested condition (TnIF_cdt_) and the control condition (TnIF_CTRL_). The frequency distribution of ΔTnIF values was plotted for both chromosomes and for each tested condition (**S6 Fig**), to identify the main peak of unaffected ΔTnIF values and its standard deviation. 2% of ΔTnIF values at each extremity were removed to avoid an influence of extreme values, the standard deviation was calculated on this distribution. Depending on the conditions tested, the standard deviation ranged from 0.049 and 0.244. The ΔTnIF values larger than 0.5 were thus selected as significant, since they correspond to 2 to 5 standard deviations from the mode, designating genes for which the TnIF value was decreased compared to the control condition.

### Infection of RAW 264.7 macrophages using the transposon mutant library

For each time of infection, an independent mini-Tn5 library was generated (complexity >10^6^) and colonies were pooled in 2YT medium, diluted in RAW 264.7 macrophage culture medium to reach a multiplicity of infection (MOI) of 1:50, and added to the macrophages, which were previously seeded in 6-well plates to a concentration of 1.5 x 10^5^ cells per well. For each time of infection, a total of sixteen 6-well plates were prepared, and thus 1.44x10^7^ cells were infected with 7.2x10^8^ bacteria which allows to cover the complexity of the library. Macrophages were then centrifuged for 10 min at 400 *g* at 4°C and subsequently incubated for 1 hour at 37°C with 5% CO_2_. The culture medium was then removed and replaced with fresh medium containing gentamicin at 50 μg/ml in order to kill extracellular bacteria, and macrophages were then further incubated for 23 hours at 37°C with 5% CO_2_. At 24 hours post-infection, culture medium was removed, each well was washed twice with phosphate-buffered saline (PBS), and macrophages were lysed using PBS–0.1% Triton X-100 for 10 min at 37°C. Macrophage lysates were then spread on 100 2YT plates with kanamycin and incubated at 37°C for 4 days in order to obtain colonies that were collected for the preparation of genomic DNA (gDNA) and sequencing of the Tn*5* insertion flanking sequences.

### Construction of deletion mutants to verify Tnseq predictions

The Δ*glpX*, Δ*wbkF*, Δ*gmd*, Δ*per*, Δ*fadA*, Δ*fadJ* and Δ*trpD* mutants were constructed in the *B*. *melitensis* 16M WT strain by triparental mating to introduce the pNPTS138 Kan^R^ plasmid (containing the upstream joined to the downstream region, generated by PCR, for the respective genes of interest for deletion) in the *B*. *melitensis* 16M Nal strain using the *E*. *coli* MT 607 (*pro-82 thi-I hsdR17* (r-m+) *supE44 recA56* pRK600) strain (described in [61]), and allelic replacement was performed as described previously for other gene deletions [24]. Δ*glpX* deletion plasmidic constructs were a gift from Amaia Zúñiga Ripa [62]. Deletion of the following genes was checked using the respective primers: the GlpXFCheck 5’-GACATTTCGCTGGAATCGATC-3’ and GlpXRCheck 5’-GGCTATATAATGTCGTCCCCATC-3’ primers for *glpX* (BMEI0726), the Forward_check_*wbkF* and Reverse_check_*wbkF* primers (see **S2 Table** for primer sequences) for *wbkF* (BMEI1426), the Forward_check_*gmd* and Reverse_check_*gmd* primers for *gmd* (BMEI1413), the Forward_check_*fadA* and Reverse_check_*fadA* primers for *fadA* (BMEII0496), the Forward_check_*fadJ* and Reverse_check_*fadJ* primers for *fadJ* (BMEII0497), the Forward_check_*trpD* and Reverse_check_*trpD* primers for *trpD* (BMEI0843). The primers used to amplify upstream and downstream regions of each gene to be deleted can also be found in **S3 Table**. The deletion of virB1-virB12 BMEII0025-BMEII0035 in the mCherry *B*. *melitensis* 16M background is described in [23].

Cultures were grown overnight with shaking at 37°C in 2YT medium and were washed twice in RPMI 1640 (Gibco Laboratories) (3500 *g*, 10 min) before inoculation in the mice.

### Brucella melitensis staining with eFluor^670^

For some histological and flow cytometry experiments, we labelled *B*. *melitensis* with eFluor^670^. Cultures (10 ml) were grown overnight as indicated above. Bacteria in 1 ml of culture were centrifuged (2 min, 7500 rpm, RT) and the pellets were washed 3 times with 1 ml of PBS, then the bacteria were incubated for 20 min at RT in the dark with eFluor^670^ dye at the final concentration of 10 μM per ml of PBS. After incubation, the bacteria were washed three times in 1 ml of PBS and once in 1 ml of RPMI before inoculation of the mice.

### RAW 264.7 macrophage infection and CFU counting

The infection protocol for performing CFU counting was identical to the one described above, except for the inoculum, which was taken from an overnight liquid culture of 0.5 ml medium/well, 10^5^ cells/well, infection with an MOI of 1:50 (50 bacteria per cell on average) in four 24-wells plates. After infection, infected macrophages were lysed at 5, 24 or 48 hours post-infection and the lysates (0.5 ml per well) were spread on 2YT plates supplemented with nalidixic acid. CFU were counted after 4 days of incubation at 37°C and expressed per ml of infected cells.

### Brucella melitensis infection in vivo

Mice were anesthetized with a cocktail of Xylazine (9 mg/kg) and Ketamine (36 mg/kg) in PBS before being inoculated intranasally with 5 × 10^6^ CFU of *B*. *melitensis* in 30 μl of RPMI. Control animals were inoculated with the same volume of RPMI. We used a mCherry-expressing wild-type (WT) 16M strain (12), mCherry expressing ΔVirB 16M strain [49] or gene deletion mutants in the wild-type *B*. *melitensis* 16M background as indicated for the infections. The infectious doses were validated by plating serial dilutions of the inoculums. At the selected time after infection, mice were sacrificed by cervical dislocation. Immediately after sacrifice, spleen and/or lung cells were collected for bacterial count, flow cytometry, and/or microscopic analyses. All infections were performed in an Animal Biosafety Level 3 facility.

For bacterial counting, organs were homogenized in PBS/0.1% X-100 Triton (Sigma-Aldrich). We performed successive serial dilutions in RPMI to obtain the most accurate bacterial count and plated them on 2YT medium. The CFU were counted after 4 days of incubation at 37°C.

### Cytofluorometric analysis

The lungs were harvested and cut into small pieces. As described previously [9], spleens were harvested, cut into small pieces and incubated for 1 hour at 37°C with a mix of 100 μg/ml of DNAse I fraction IX (Sigma-Aldrich) and 1.6 mg/ml of collagenase (400 Mandl U/ml). The cells were then washed, filtered and incubated with saturating doses of purified 2.4G2 (anti-mouse Fc receptor, ATCC) in 200 μl PBS, 0.2% BSA, 0.02% NaN3 (FACS buffer) for 20 min at 4°C to prevent antibody (Ab) binding to the Fc receptor.

3–5x10^6^ cells were stained on ice with various fluorescent mAb combinations in FACS buffer. We acquired the following mAbs and reagents from BD Biosciences: BV421-coupled E50-2440 (anti-Siglec-F), BV421-coupled AL-21 (anti Ly-6C), BV421-coupled 1A8 (anti-Ly-6G), BV421-coupled T45-2342 (anti-F4/80), BV421-coupled M1/70 (anti-CD11b), BV421-coupled 1A8 (anti-Ly6G), fluorescein (FITC)-coupled HL3 (anti-CD11c), biotin-coupled 2G9 (anti-MHCII, I-A/I-E), fluorescein (FITC)-coupled streptavidin. The cells were analyzed on a BD FacsVerse flow cytometer. Dead cells and debris were eliminated from the analysis according to size and scatter.

### Immunofluorescence microscopy of tissues

Lungs were fixed for 20 minutes at RT in 2% paraformaldehyde (PFA). Then, lungs were placed under a vacuum until no air was present in the lungs in 2% PFA for 2 hours. After fixation, lungs were incubated overnight at 4°C in a 20% PBS-sucrose solution. Tissues were then embedded in Tissue-Tek OCT compound (Sakura), frozen in liquid nitrogen, and cryostat sections (5 μm) were prepared. For staining, tissue sections were rehydrated in PBS and incubated in a PBS solution containing 1% blocking reagent (Boehringer) (PBS-1% BR) for 20 minutes before incubation overnight in PBS-1% BR containing the DAPI nucleic acid stains Alexa Fluor 350 and 488 phalloidin (Molecular Probes). Slides were mounted in Fluoro-Gel medium (Electron Microscopy Sciences, Hatfield, PA). Labelled tissue sections were visualized with an Axiovert M200 inverted microscope (Zeiss, Iena, Germany) equipped with AxioVision program and a high-resolution monochrome camera (AxioCam HR, Zeiss). Images (1384x1036 pixels, 0.16 μm/pixel) were acquired sequentially for each fluorochrome with A-Plan 10x/0.25 NA and LD-Plan-NeoFluar 63x/0.75 NA dry objectives and recorded as 8-bit grey-level *.zvi files. In order to determine the number of infected cells per unit area, we used the scaling tool of the AxioVision program from Zeiss to measure the area of a viewing field and then divided the number of infected cells counted by the number of fields observed. For all microscopy analysis of *in vivo* experiments, at least 3 slides were analyzed per organ from 3 different animals and the entire experiment is carried out twice independently.

### Confocal microscopy

Confocal analyses were performed using an LSM780 confocal system fitted on an Observer Z 1 inverted microscope equipped with an alpha Plan Apochromat 63x/1.46 NA oil immersion objective (Zeiss, Iena, Germany). DAPI was excited using a 405 nm blue diode, and emission was detected using a band-pass filter (410–480 nm). The 488 nm excitation wavelength of the Argon/2 laser was used in combination with a band-pass emission filter (BP500-535 nm) to detect Alexa Fluor 488 phalloidin. The 543 nm excitation wavelength of the HeNe1 laser and a band-pass emission filter (BP580-640 nm) were used for the red fluorochrome mCherry. The 633 nm excitation wavelength of the HeNe2 laser and a band-pass emission filter (BP660-695 nm) were used for far-red fluorochromes such as eFluor^670^. To ensure optimal separation of the fluorochromes, blue & red signals were acquired simultaneously in one track and green & far-red signals were acquired in a second track. The electronic zoom factor and stack depth were adjusted to the region of interest while keeping image scaling constant (x-y: 0.066 micron, z: 0.287 micron). A line average of 4 was used and datasets were stored as 8-bit proprietary *.czi files. The images were displayed using Zen2012 software (Zeiss) with linear manual contrast adjustment and exported as 8-bit uncompressed *.TIF images. The figures, representing single optical sections across the region of interest, were prepared using the Canvas program.

### Purification and transmission electron microcopy analysis of alveolar macrophages

Pulmonary alveolar macrophages were obtained by homogenization and filtration of lungs, density gradient centrifugation (1.085 g/cm^3^ nycodenz, 1700xg during 30 min) and CD11c specific Magnetic associated cell sorting (MACS) by using MiniMACS, MS column and CD11c+ magnetic beads (Miltenyl Biotec).

Purified alveolar macrophages were fixed 2 hours in 2% glutaraldehyde in 0.1 M cacodylate buffer at 4°C, washed 3 times (4000xg, 5 min) with 0.2 M cacodylate buffer then post-fixed in 2% osmium tetroxide in 0.1 M cacodylate buffer for 1 hour at RT. After 3 washes, samples were serially dehydrated in ethanol (30% EtOH first 5 min, followed by 10 min–the same for 50%, 70%, 85%, 100% EtOH). Propylene oxide was then added 4x5 min at RT and progressively embedded in epoxy resin (Agar 100 resin; Agar Scientific, United Kingdom) (75,25, 50:50 and then 25:75% if propylene oxide/resin). Ultrathin 50-nm sections were obtained, mounted on copper-Formvar-carbon grids (EMS, United Kingdom), and stained with uranyl acetate and lead citrate by standard procedures. A Tecnai 10 electron microscope (FEI, Eindhoven, The Netherlands) was used for observations, and images were captured with a Veleta charge-coupled-device (CCD) camera and processed using the AnalySIS and Adobe Photoshop software programs.

### Statistical analysis

For comparisons between two groups, we used a (Wilcoxon-)Mann-Whitney test provided by the GraphPad Prism software to statistically analyze our results. For comparisons between more than two groups, we used a One-Way ANOVA with Kruskal-Wallis post-test. Values of p < 0.05 were considered to represent a significant difference: *p < 0.05, **p < 0.01, ***p < 0.001, ****p < 0.0001.

## Supporting information

S1 Fig Alveolar macrophages are the main cells infected with *B*. *melitensis* in the lung. Wild-type C57BL/6 mice (n = 5) received PBS (control mice) or 5x10^6^ CFU mCherry-expressing *B*. *melitensis* labelled with eFluor^670^ in PBS intranasally. Mice were sacrificed at 48 hours post-infection. The lungs were harvested, and the cells were isolated and then analyzed by flow cytometry for the expression of FSC, eFluor^670^, mCherry, Siglec-F, CD11b, CD11c, F4/80, Ly6C, Ly6G and MHCII as indicated. **A.** Gating strategy. Numbers indicate the percentage of eFluor^670+^ cells among the total cells and the percentage of mCherry^high^ cells among the eFluor^670+^ cells. **B.** Cell surface phenotype of eFluor^670+^ cells. These results are representative of three independent experiments.
(TIF)

S2 Fig eFluor^670^ labelling of mCherry-expressing *Brucella* identified distinct bacterial states. **A:** Schematic representation of unipolar growth of eFluor^670^-labelled mCherry-*Brucella*. As eFluor^670^ does not move on the bacterial surface, the newly formed bacterium, called the daughter cell, loses the eFluor^670^ labelling during unipolar growth, at least at the second generation, and therefore it can be identified by fluorescent microscopy. The lack of mCherry expression is correlated to bacteria death. **B:** C57BL/6 mice (n = 5) were infected with 5x10^6^ CFU of mCherry-expressing *B*. *melitensis* labelled with eFluor^670^ and sacrificed at the indicated time. Lungs were collected and analyzed by fluorescent microscopy for the labelling of DAPI, phalloidin, mCherry and eFluor^670^. Data shown are representative images of infected cells. The panels are color-coded with the text for mCherry and eFluor^670^.
(TIF)

S3 Fig Morphology of living, stressed and dead *B*. *melitensis*. **A**: Comparison of the growth of *B*. *melitensis* in 2YT rich medium and in Plommet-erythritol minimal medium. The bacteria were grown for 72 hours at 37°C and the OD was measured every 30 min in a Bioscreen system. The deviation was obtained from two independent experiments. **B, C:** Transmission electron microscopy analysis of control condition (incubated for 24 hours in 2YT rich medium), stressed (incubated for 24 hours in Plommet-erythritol minimal medium) and heat killed (incubated for 30 min at 80°C) *B*. *melitensis*.
(TIF)

S4 Fig Frequency distribution of TnIF values of genes from chromosomes I and II of *B*. *melitensis* in 2YT rich medium condition. **A, B:** The TnIF values for each gene from chromosome I (**A**) and chromosome II (**B**) are represented by classes of 0.1. The blue histogram shows the distribution for TnIF values for all genes of *B*. *melitensis* per chromosome. The orange line separated genes that are considered as unaltered in 2YT rich medium to the others. **C**: The TnIF of each gene of the *B*. *melitensis* genome of the control Tn-seq on 2YT plates sequenced with an Illumina HiSeq was compared to the TnIF of an independent repeated Tn-Seq on 2YT sequenced with an Illumina NextSeq sequencing. A Pearson correlation coefficient was calculated and equal to 0.98.
(TIF)

S5 Fig Frequency distribution of ΔTnIF values of genes from chromosomes I and II of *B*. *melitensis* for each tested condition in RAW 264.7 cell line and mice. The ΔTnIF values were represented by class of 0.2. The blue histogram shows the distribution for ΔTnIF values for all genes that are untouched in the control 2YT condition. The red color represents the distribution for ΔTnIF values without 2% of number of genes at each extremity. SD means standard deviation.
(PDF)

S6 Fig Analysis of Tn-seq data under *in vitro* and *in vivo* conditions. **A, B, C:** Data shown are the kinetics of TnIF values (in 2YT rich medium (CTRL) and at 5, 24, 48, 120 hours post-infection in the lung) for (**A**) all genes, (**B**) genes implicated in LPS biosynthesis and (**C**) genes implicated in the type IV secretion system (T4SS) that were identified as LF and VLF (ΔTnIF > 0.5) at 120 hours post-infection in lungs of wild-type mice. Red line and red number indicate the median TnIF value of all LF and VLF genes. Dashed blue line indicates the mode TnIF value of all *B*. *melitensis* genes in 2YT.
(TIF)

S7 Fig Identification of immune effector mechanisms controlling the early multiplication of *B*. *melitensis* in lungs of wild-type mice. Wild-type (wt), IFNγR^-/-^, TNFR1^-/-^, IL-17RA^-/-^, IL-4^-/-^ C57BL/6 mice and wild-type asthmatic mice were infected intranasally with a dose of 5×10^6^ CFU of mCherry-*B*. *melitensis*. Mice were sacrificed at the indicated times, the lungs were harvested and analyzed for CFU count by flow cytometry. The data represent (**A**) the CFU count per g/lung and (**B**) the percentage of mCherry^high^ cells among the eFluor^+^ lung cells per individual mice as determined by flow cytometry. Gray bars represent the median. Significant differences between wt and the indicated groups are marked with asterisks: *p < 0.1, **p < 0.01, ***p < 0.001, in a One-Way ANOVA with Kruskal-Wallis post-test.
(TIF)

S1 Table List of essentiality and TnIF values for all *B*. *melitensis* genes in the 2YT rich medium condition. Genes are listed by chromosome and in descending order of TnIF value. Genes are essential (ES), if the R100 value drops to 0 at least once.
(XLSX)

S2 Table List of attenuated *B*. *melitensis* genes in mice in the lung (5h, 24h, 48h, 120h) and RAW 264.7 (24h) conditions. Genes displaying a decrease of at least 0.5 TnIF compared to the TnIF of the gene in the 2YT rich medium (CTRL) condition are grouped by functional categories using the eggnog public database [63] and associated with their TnIF values for each condition.
(XLSX)

S3 Table List of primers used in the construction of deletion mutants. (DOCX)
