# Supplementary figures and images for: Genome-wide analysis of Brucella melitensis genes required throughout intranasal infection in mice
Source: PLoS Pathog. 2022 Jun 30;18(6):e1010621. doi: 10.1371/journal.ppat.1010621 (PMC9246152; doi:10.1371/journal.ppat.1010621)

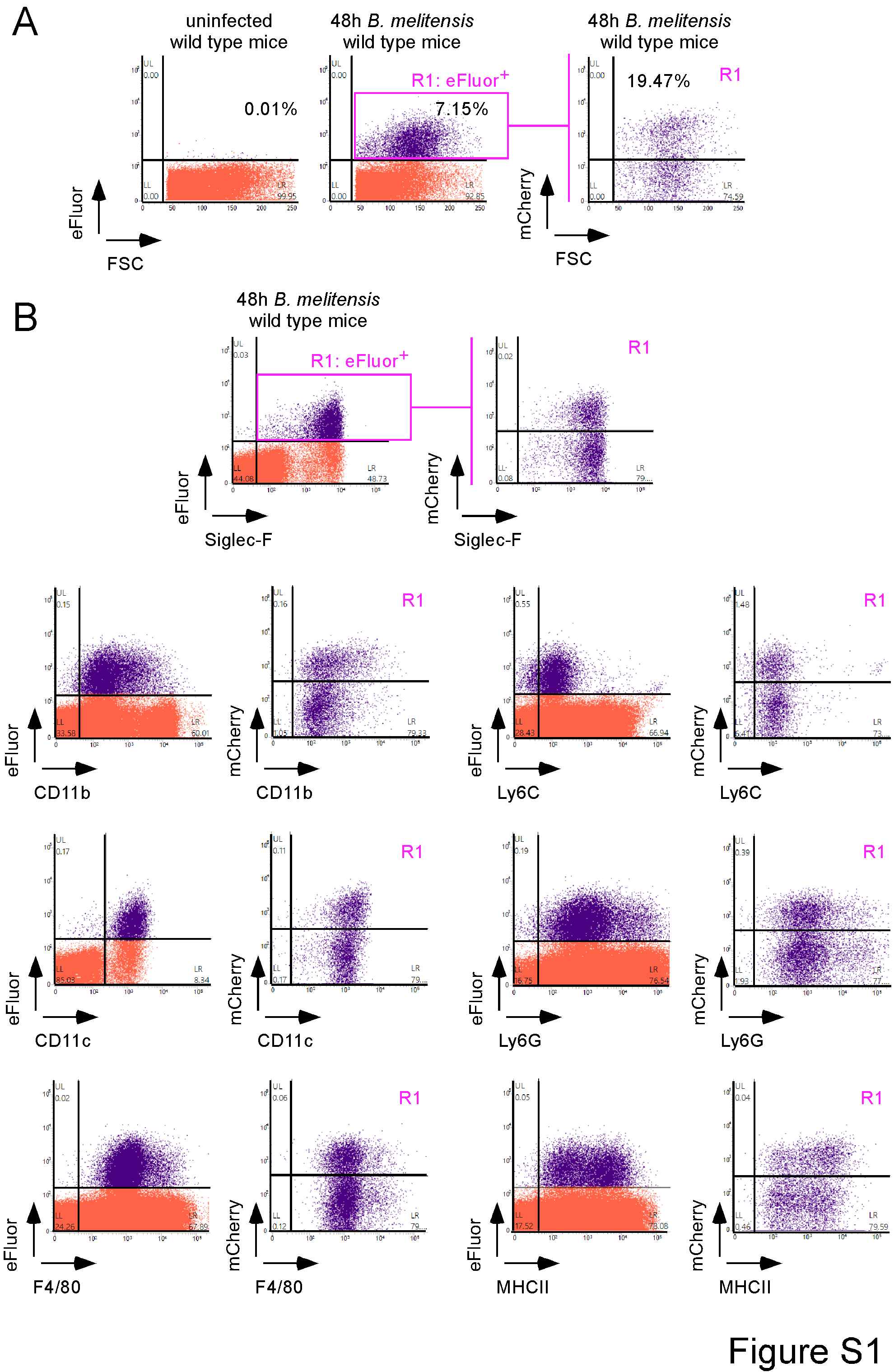

Supplement: S1 Fig — Wild-type C57BL/6 mice (n = 5) received PBS (control mice) or 5x10^6^ CFU mCherry-expressing B. melitensis labelled with eFluor670 in PBS intranasally. Mice were sacrificed at 48 hours post-infection. The lungs were harvested, and the cells were isolated and then analyzed by flow cytometry for the expression of FSC, eFluor670, mCherry, Siglec-F, CD11b, CD11c, F4/80, Ly6C, Ly6G and MHCII as indicated. A. Gating strategy. Numbers indicate the percentage of eFluor670+ cells among the total cells and the percentage of mCherryhigh cells among the eFluor670+ cells. B. Cell surface phenotype of eFluor670+ cells. These results are representative of three independent experiments. (TIF) [file ppat.1010621.s001.tif]

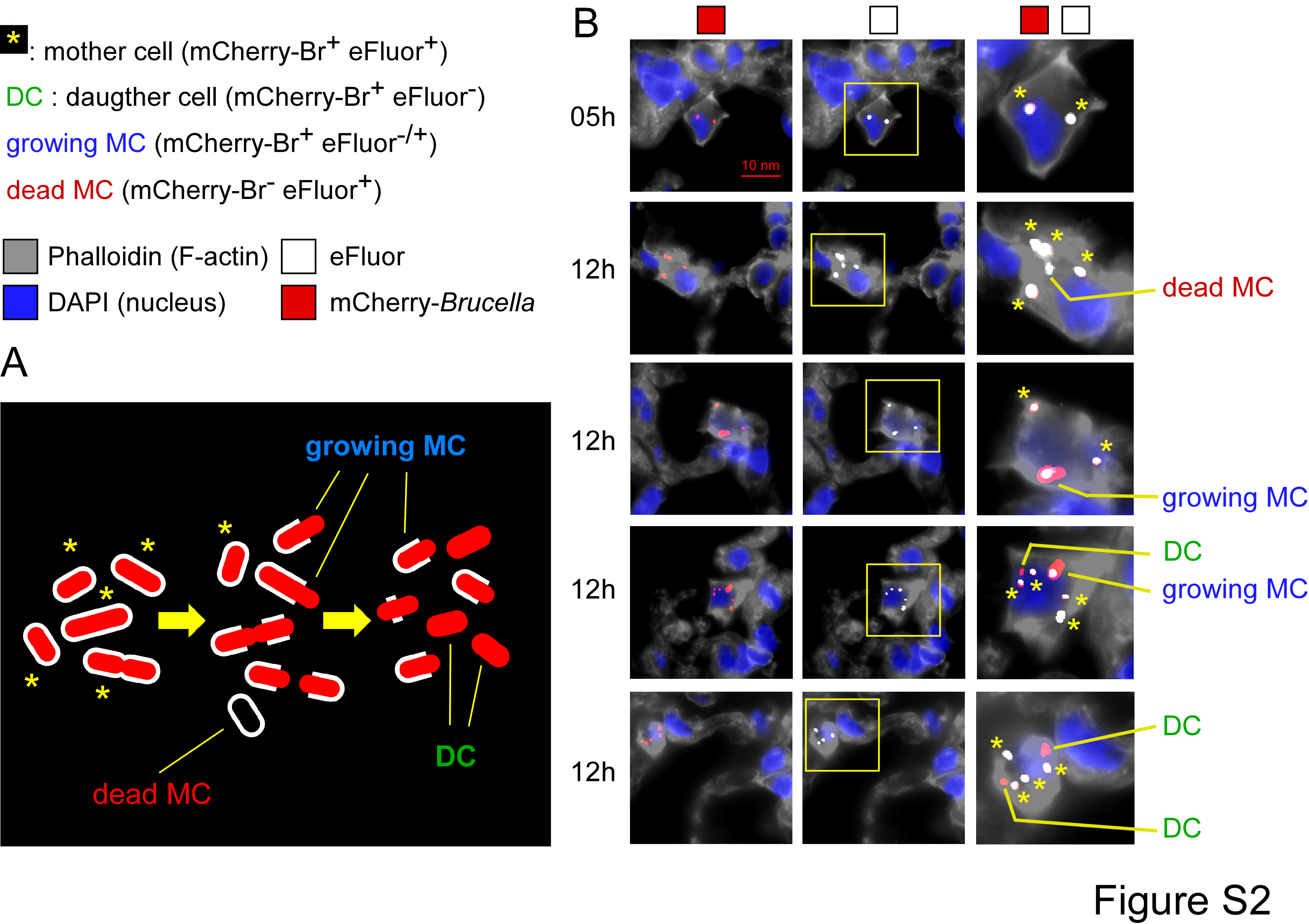

Supplement: S2 Fig — A: Schematic representation of unipolar growth of eFluor670-labelled mCherry-Brucella. As eFluor670 does not move on the bacterial surface, the newly formed bacterium, called the daughter cell, loses the eFluor670 labelling during unipolar growth, at least at the second generation, and therefore it can be identified by fluorescent microscopy. The lack of mCherry expression is correlated to bacteria death. B: C57BL/6 mice (n = 5) were infected with 5x10^6^ CFU of mCherry-expressing B. melitensis labelled with eFluor670 and sacrificed at the indicated time. Lungs were collected and analyzed by fluorescent microscopy for the labelling of DAPI, phalloidin, mCherry and eFluor670. Data shown are representative images of infected cells. The panels are color-coded with the text for mCherry and eFluor670. (TIF) [file ppat.1010621.s002.tif]

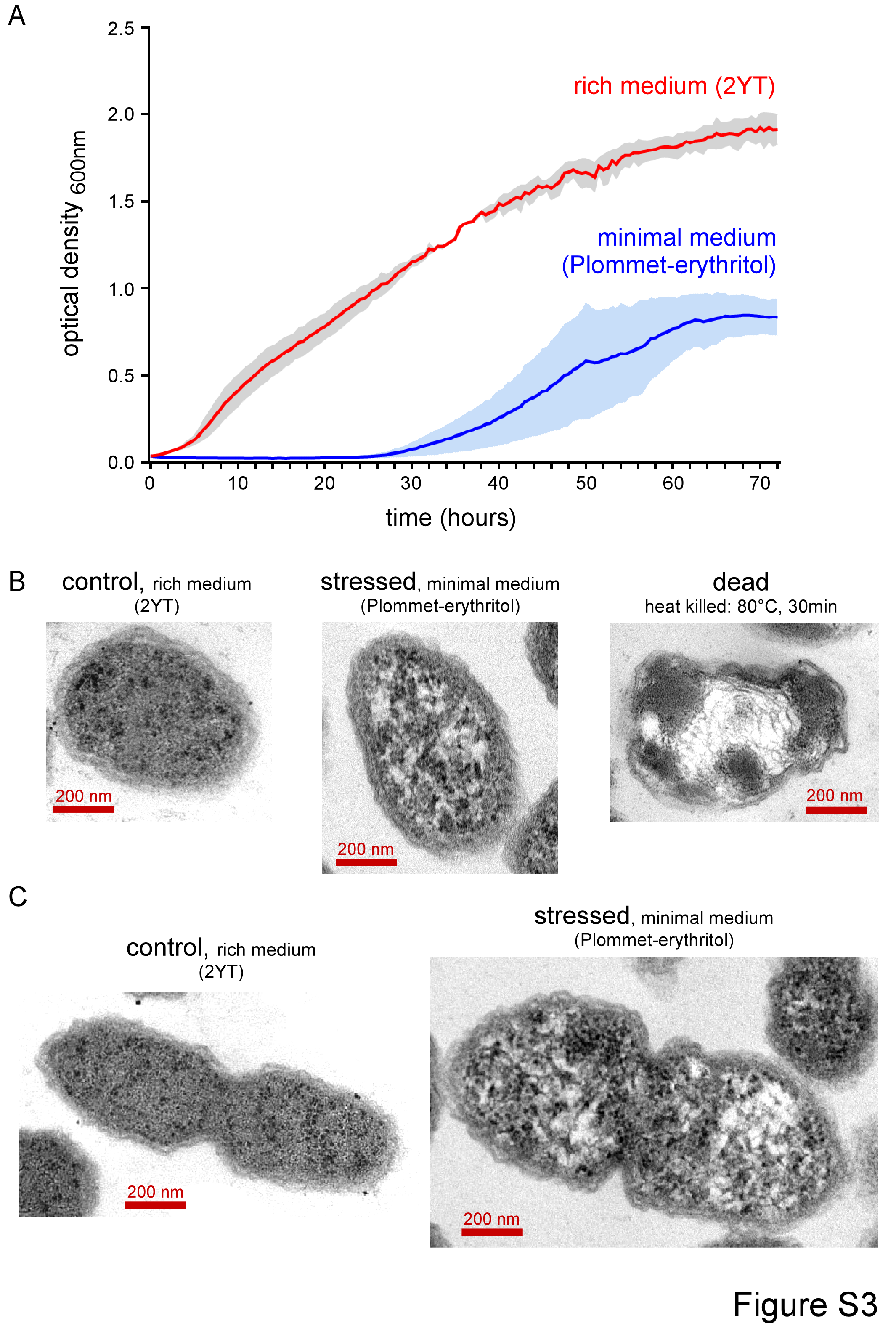

Supplement: S3 Fig — A: Comparison of the growth of B. melitensis in 2YT rich medium and in Plommet-erythritol minimal medium. The bacteria were grown for 72 hours at 37°C and the OD was measured every 30 min in a Bioscreen system. The deviation was obtained from two independent experiments. B, C: Transmission electron microscopy analysis of control condition (incubated for 24 hours in 2YT rich medium), stressed (incubated for 24 hours in Plommet-erythritol minimal medium) and heat killed (incubated for 30 min at 80°C) B. melitensis. (TIF) [file ppat.1010621.s003.tif]

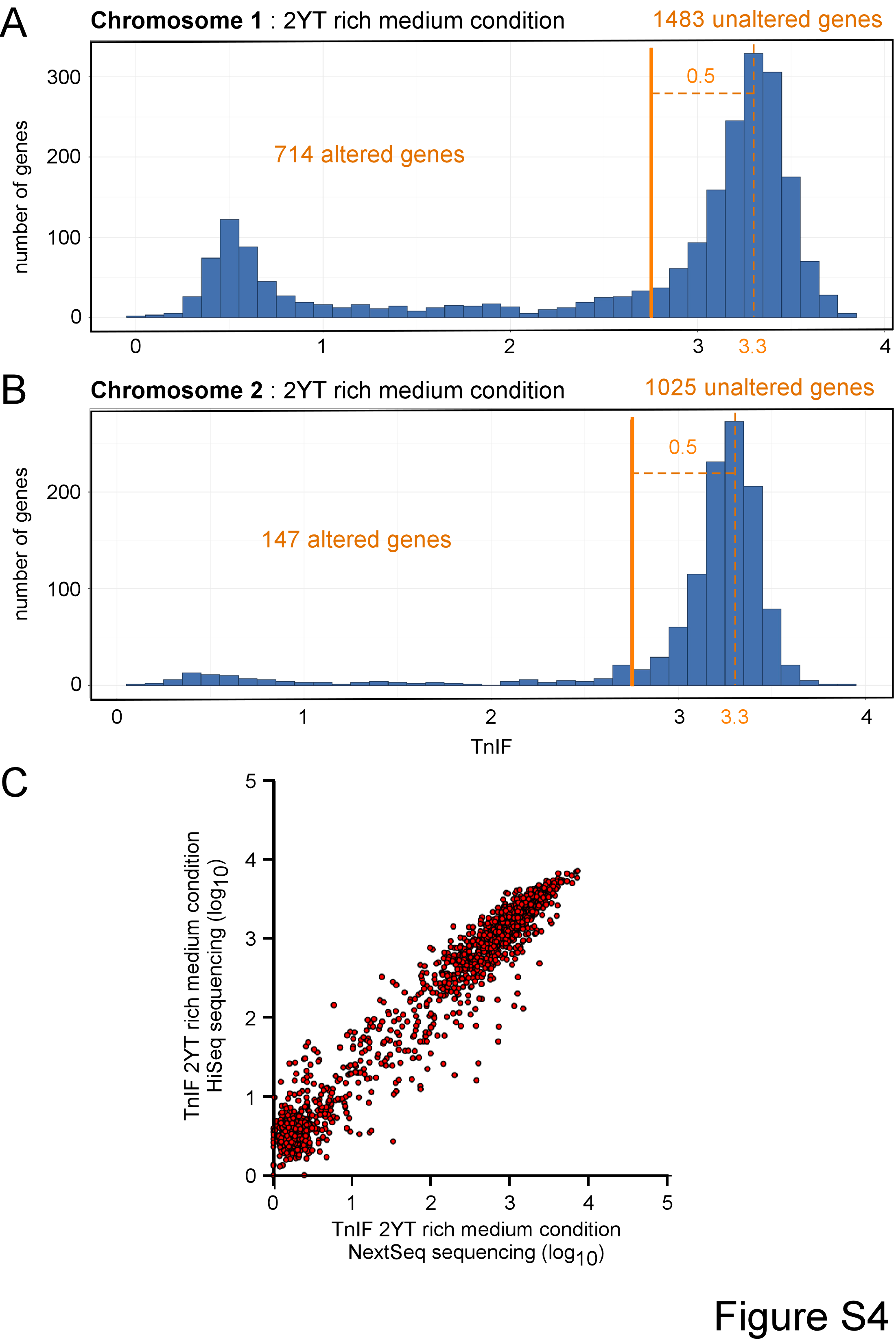

Supplement: S4 Fig — A, B: The TnIF values for each gene from chromosome I (A) and chromosome II (B) are represented by classes of 0.1. The blue histogram shows the distribution for TnIF values for all genes of B. melitensis per chromosome. The orange line separated genes that are considered as unaltered in 2YT rich medium to the others. C: The TnIF of each gene of the B. melitensis genome of the control Tn-seq on 2YT plates sequenced with an Illumina HiSeq was compared to the TnIF of an independent repeated Tn-Seq on 2YT sequenced with an Illumina NextSeq sequencing. A Pearson correlation coefficient was calculated and equal to 0.98. (TIF) [file ppat.1010621.s004.tif]

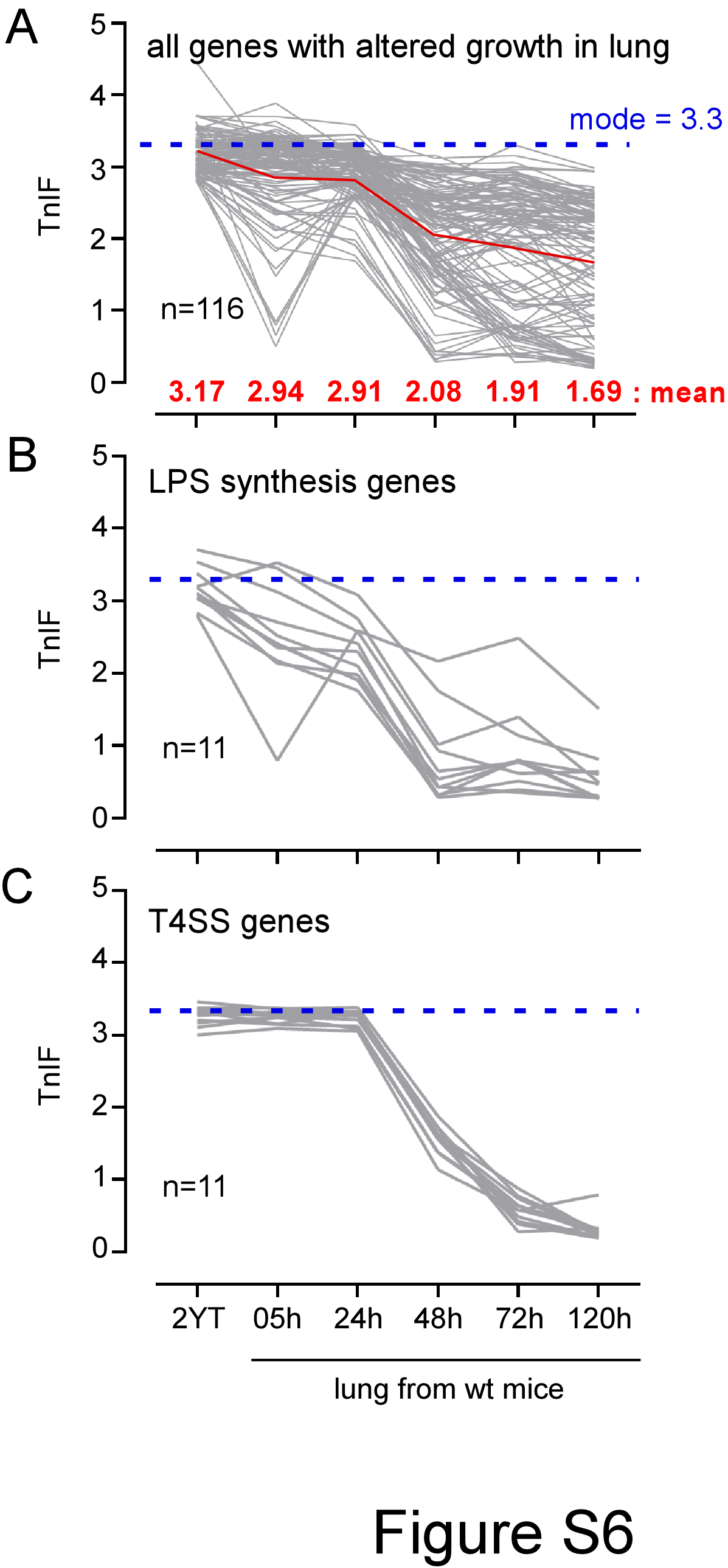

Supplement: S6 Fig — A, B, C: Data shown are the kinetics of TnIF values (in 2YT rich medium (CTRL) and at 5, 24, 48, 120 hours post-infection in the lung) for (A) all genes, (B) genes implicated in LPS biosynthesis and (C) genes implicated in the type IV secretion system (T4SS) that were identified as LF and VLF (ΔTnIF > 0.5) at 120 hours post-infection in lungs of wild-type mice. Red line and red number indicate the median TnIF value of all LF and VLF genes. Dashed blue line indicates the mode TnIF value of all B. melitensis genes in 2YT. (TIF) [file ppat.1010621.s006.tif]

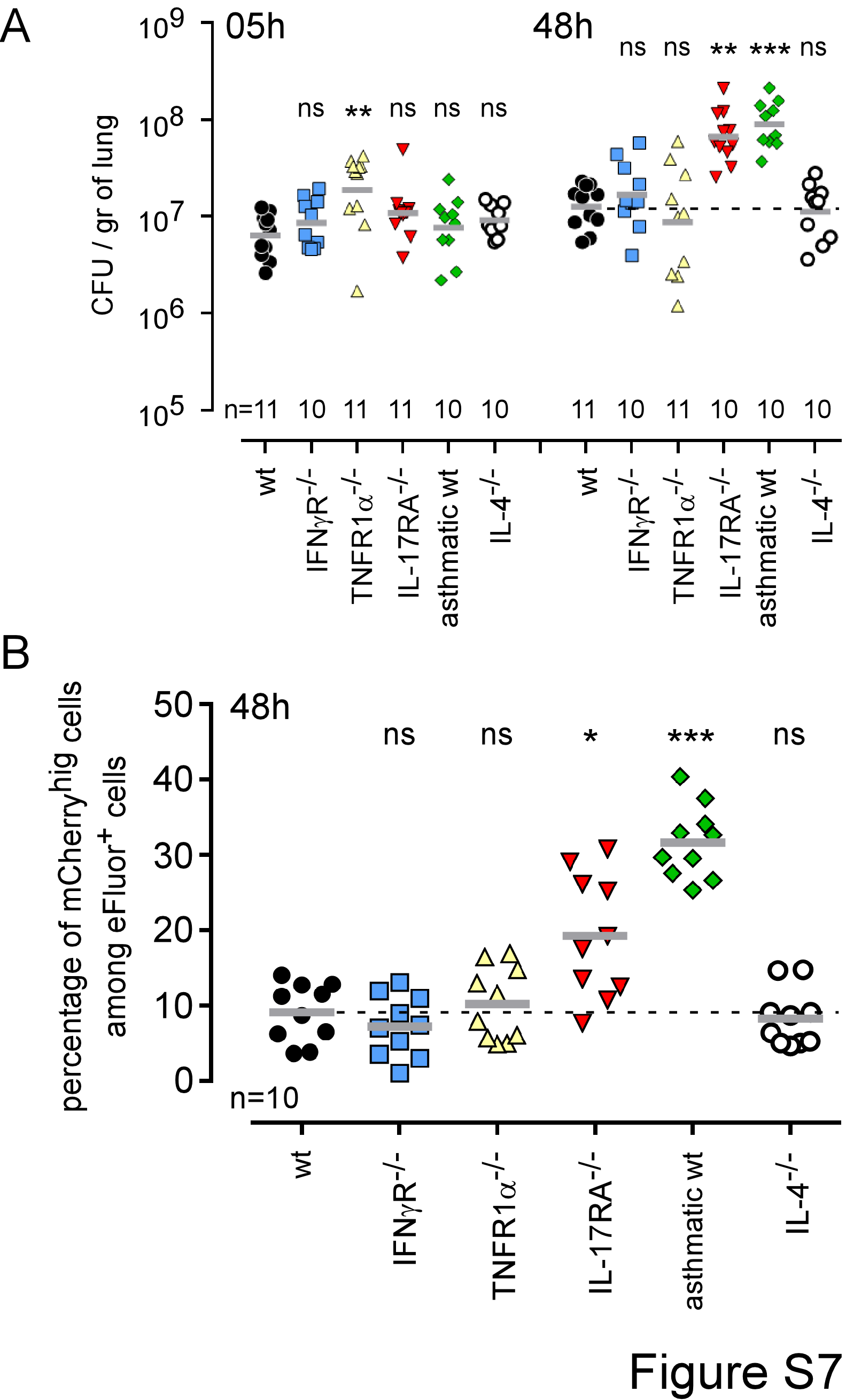

Supplement: S7 Fig — Wild-type (wt), IFNγR-/-, TNFR1-/-, IL-17RA-/-, IL-4-/- C57BL/6 mice and wild-type asthmatic mice were infected intranasally with a dose of 5×10^6^ CFU of mCherry-B. melitensis. Mice were sacrificed at the indicated times, the lungs were harvested and analyzed for CFU count by flow cytometry. The data represent (A) the CFU count per g/lung and (B) the percentage of mCherryhigh cells among the eFluor+ lung cells per individual mice as determined by flow cytometry. Gray bars represent the median. Significant differences between wt and the indicated groups are marked with asterisks: *p < 0.1, **p < 0.01, ***p < 0.001, in a One-Way ANOVA with Kruskal-Wallis post-test. (TIF) [file ppat.1010621.s007.tif]
