# Supplementary material for: Genome-wide analysis of Brucella melitensis genes required throughout intranasal infection in mice
Source: PLoS Pathog. 2022 Jun 30;18(6):e1010621. doi: 10.1371/journal.ppat.1010621 (PMC9246152; doi:10.1371/journal.ppat.1010621)
Supplement: S5 Fig — The ΔTnIF values were represented by class of 0.2. The blue histogram shows the distribution for ΔTnIF values for all genes that are untouched in the control 2YT condition. The red color represents the distribution for ΔTnIF values without 2% of number of genes at each extremity. SD means standard deviation. (PDF) [file ppat.1010621.s005.pdf]

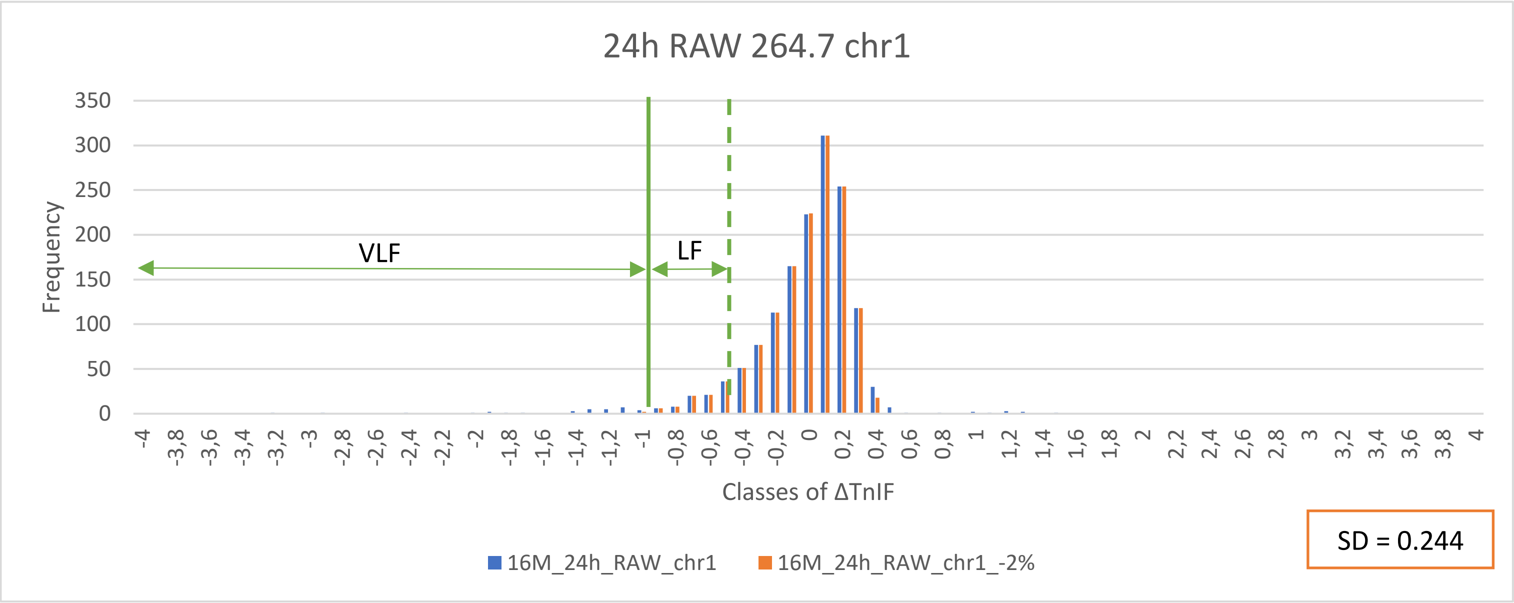
24h RAW 264.7 chr1
Frequency
VLF
LF
Classes of ΔTnIF
16M_24h_RAW_chr1
16M_24h_RAW_chr1_-2%
SD = 0.244

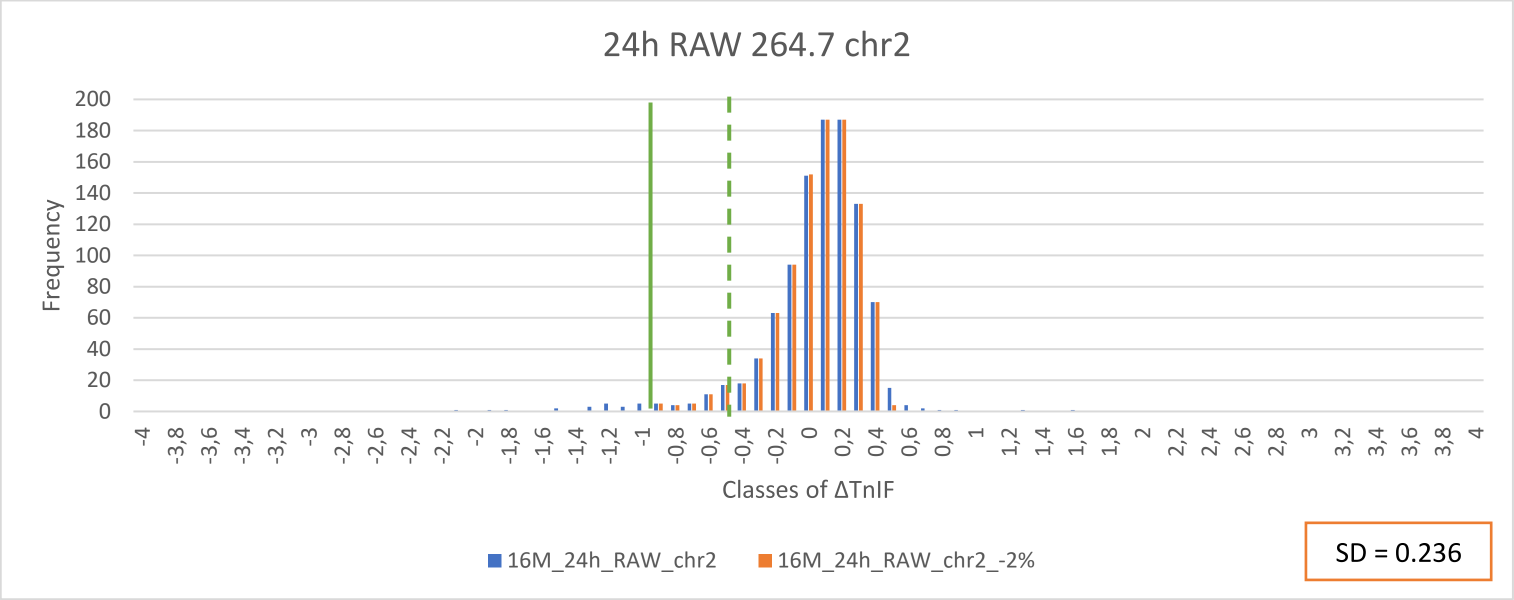
24h RAW 264.7 chr2
Frequency
Classes of ΔTnIF
16M_24h_RAW_chr2
16M_24h_RAW_chr2_-2%
SD = 0.236

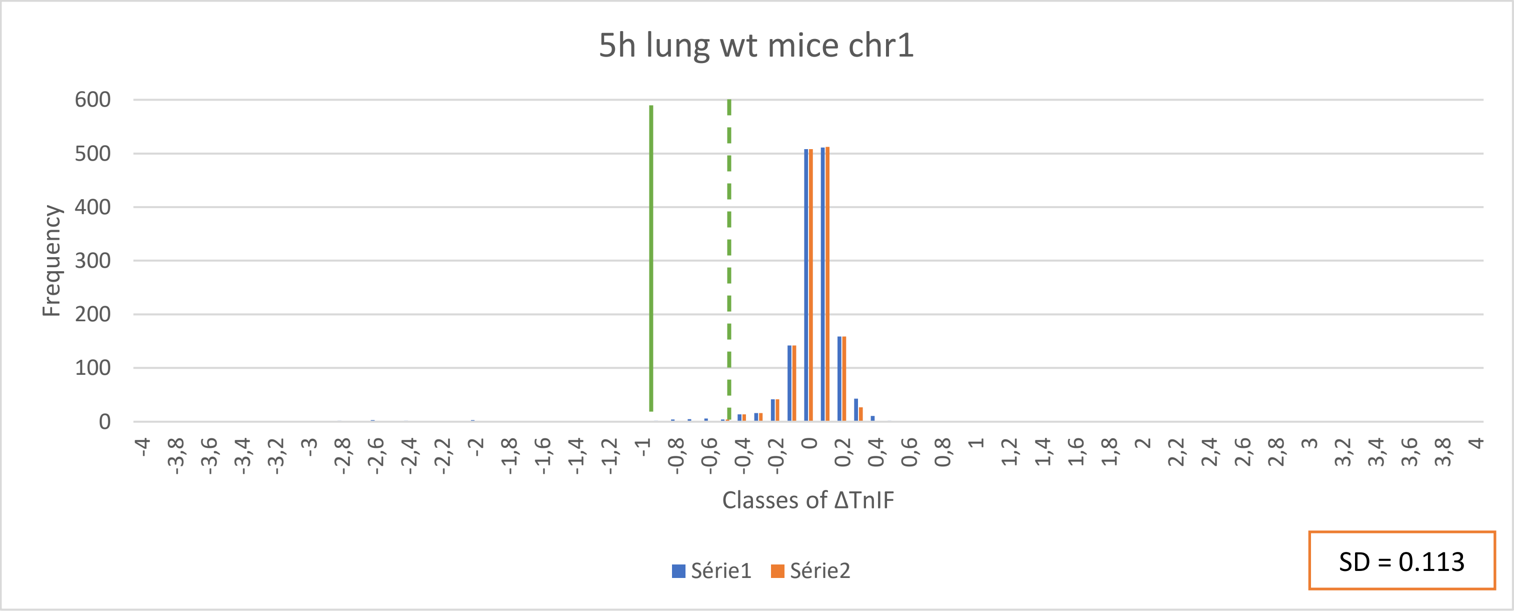
5h lung wt mice chr1
Frequency
Classes of ΔTnIF
Série1
Série2
SD = 0.113

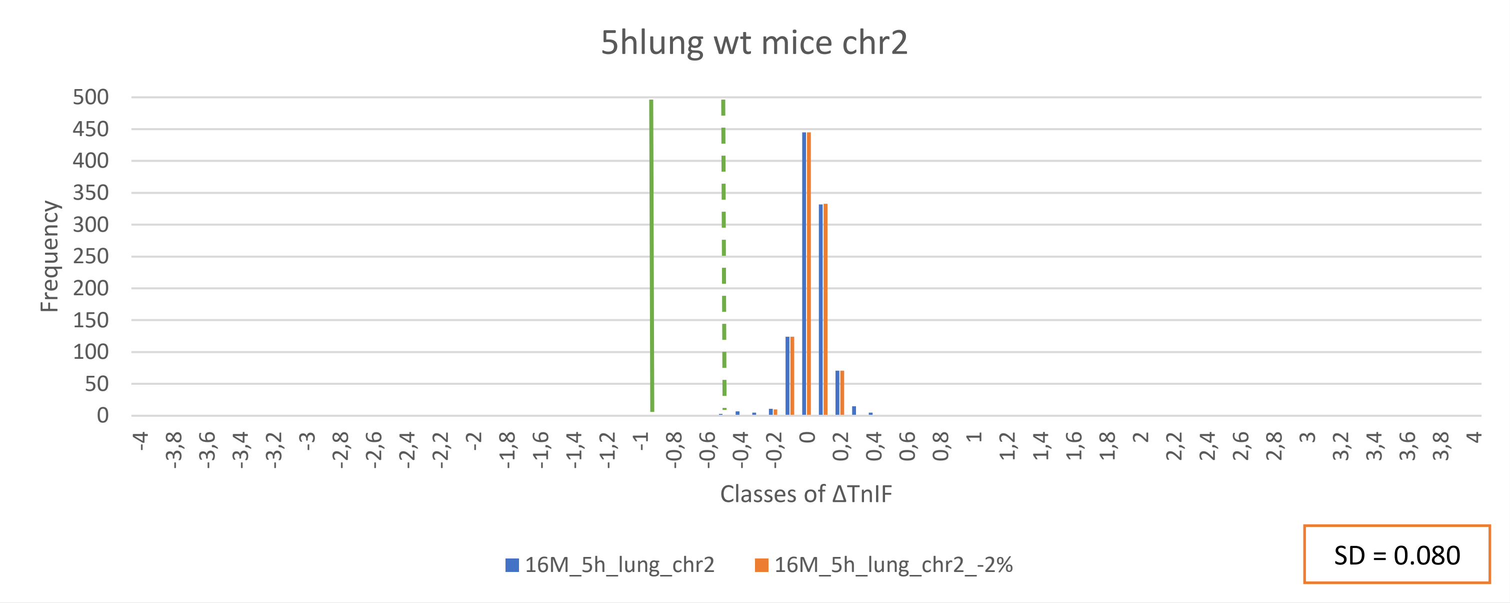
5hlung wt mice chr2
Frequency
Classes of ΔTnIF
16M_5h_lung_chr2
16M_5h_lung_chr2_-2%
SD = 0.080

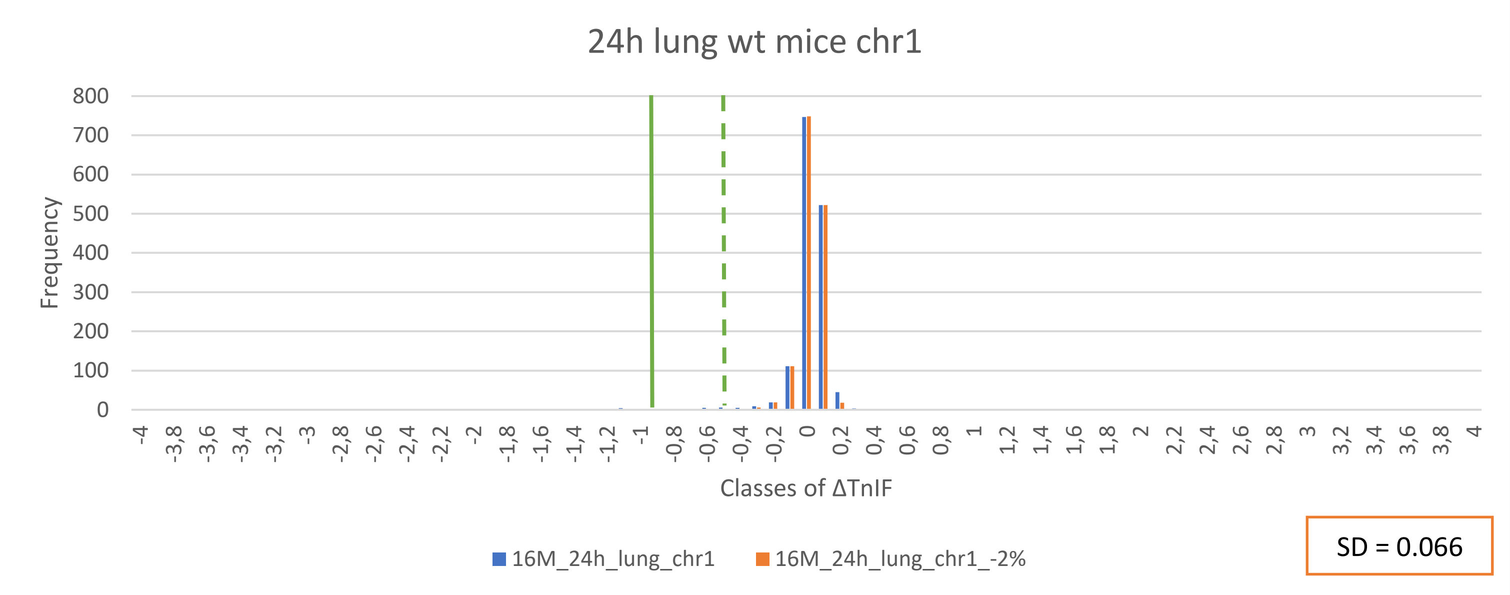
24h lung wt mice chr1
Frequency
Classes of ΔTnIF
16M_24h_lung_chr1
16M_24h_lung_chr1_-2%
SD = 0.066

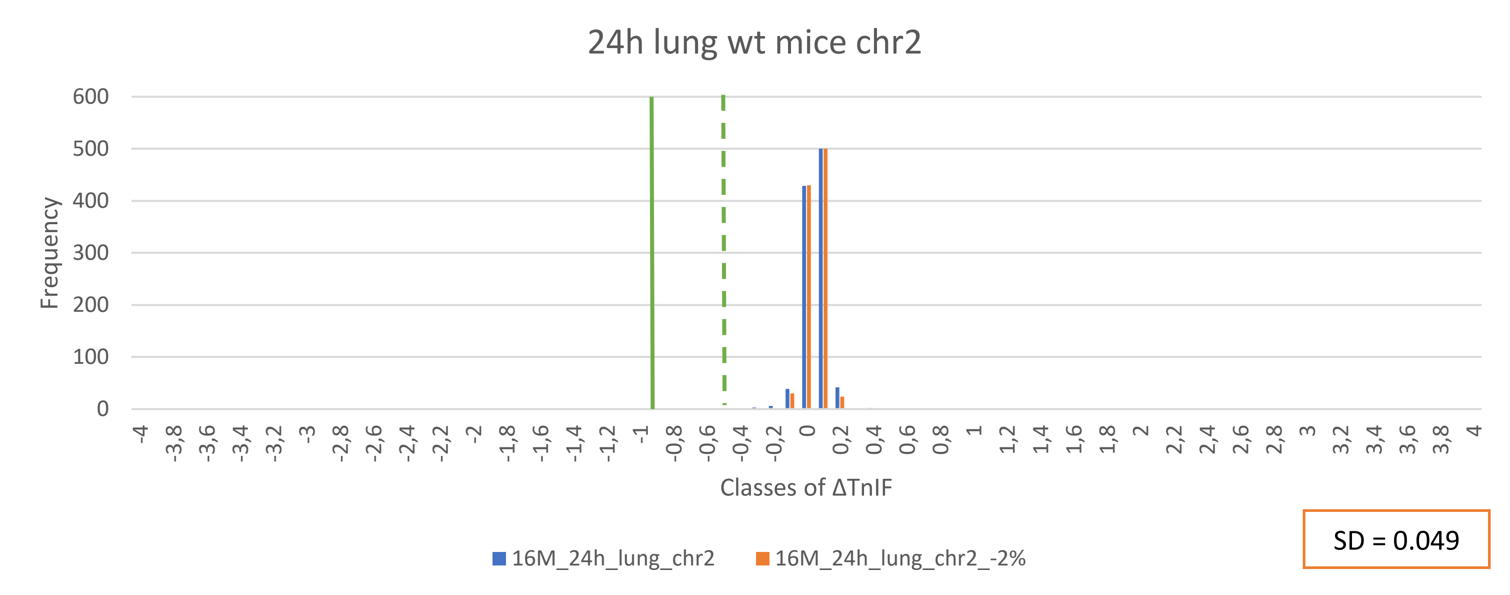
24h lung wt mice chr2
Frequency
Classes of ΔTnIF
16M_24h_lung_chr2
16M_24h_lung_chr2_-2%
SD = 0.049

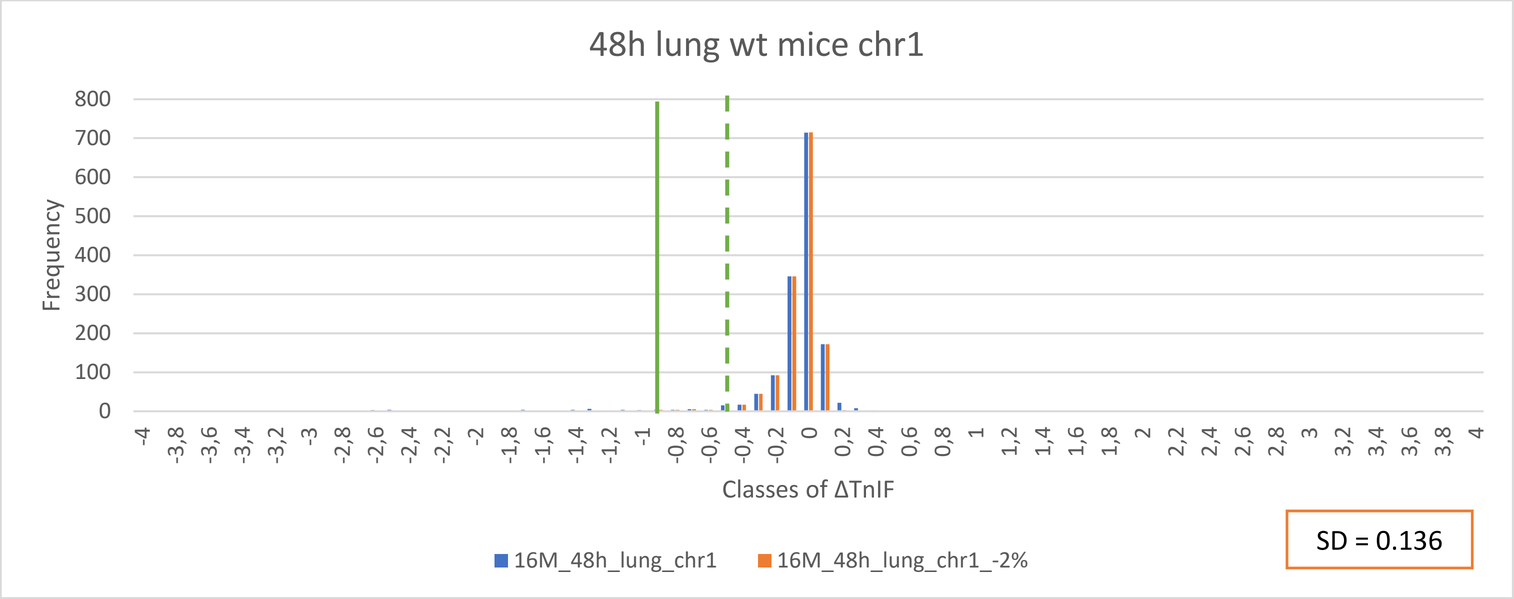
48h lung wt mice chr1
Frequency
800
700
600
500
400
300
200
100
0
-4 -3,8 -3,6 -3,4 -3,2 -3 -2,8 -2,6 -2,4 -2,2 -2 -1,8 -1,6 -1,4 -1,2 -1 -0,8 -0,6 -0,4 -0,2 0 0,2 0,4 0,6 0,8 1 1,2 1,4 1,6 1,8 2 2,2 2,4 2,6 2,8 3 3,2 3,4 3,6 3,8 4
Classes of ΔTnIF
16M_48h_lung_chr1
16M_48h_lung_chr1_-2%
SD = 0.136

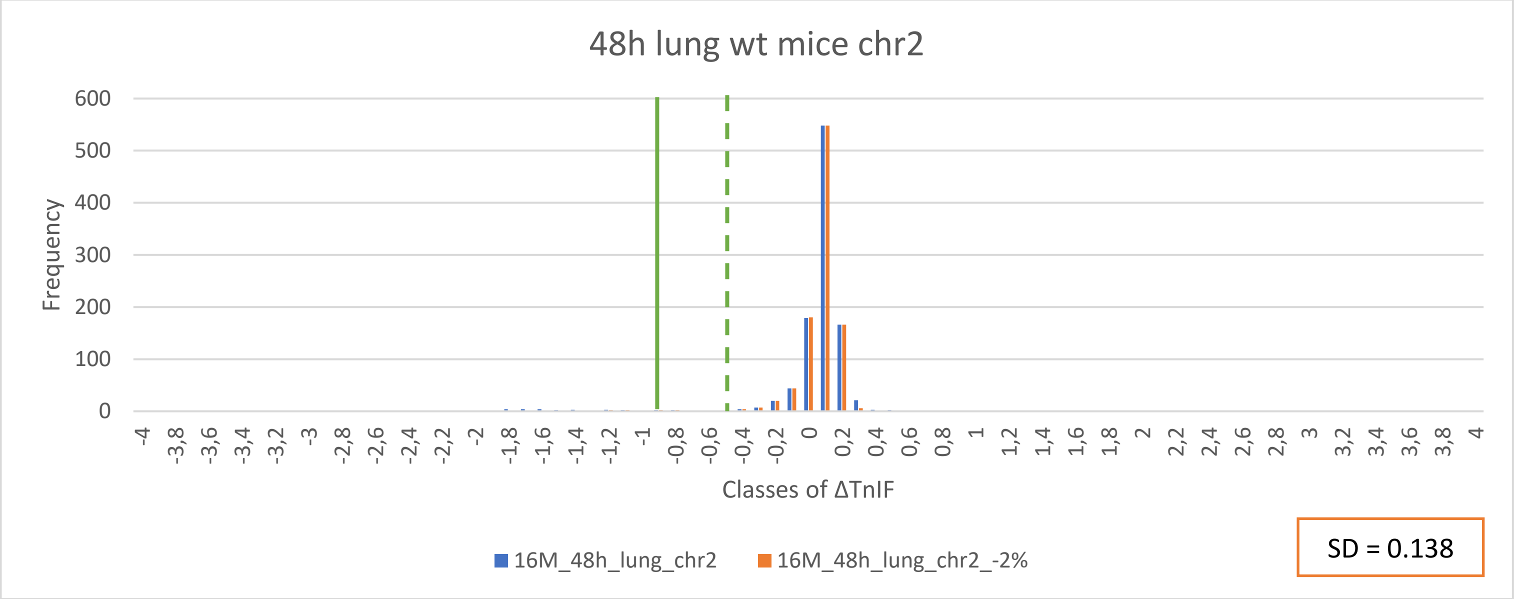
48h lung wt mice chr2
Frequency
600
500
400
300
200
100
0
-4 -3,8 -3,6 -3,4 -3,2 -3 -2,8 -2,6 -2,4 -2,2 -2 -1,8 -1,6 -1,4 -1,2 -1 -0,8 -0,6 -0,4 -0,2 0 0,2 0,4 0,6 0,8 1 1,2 1,4 1,6 1,8 2 2,2 2,4 2,6 2,8 3 3,2 3,4 3,6 3,8 4
Classes of ΔTnIF
16M_48h_lung_chr2
16M_48h_lung_chr2_-2%
SD = 0.138

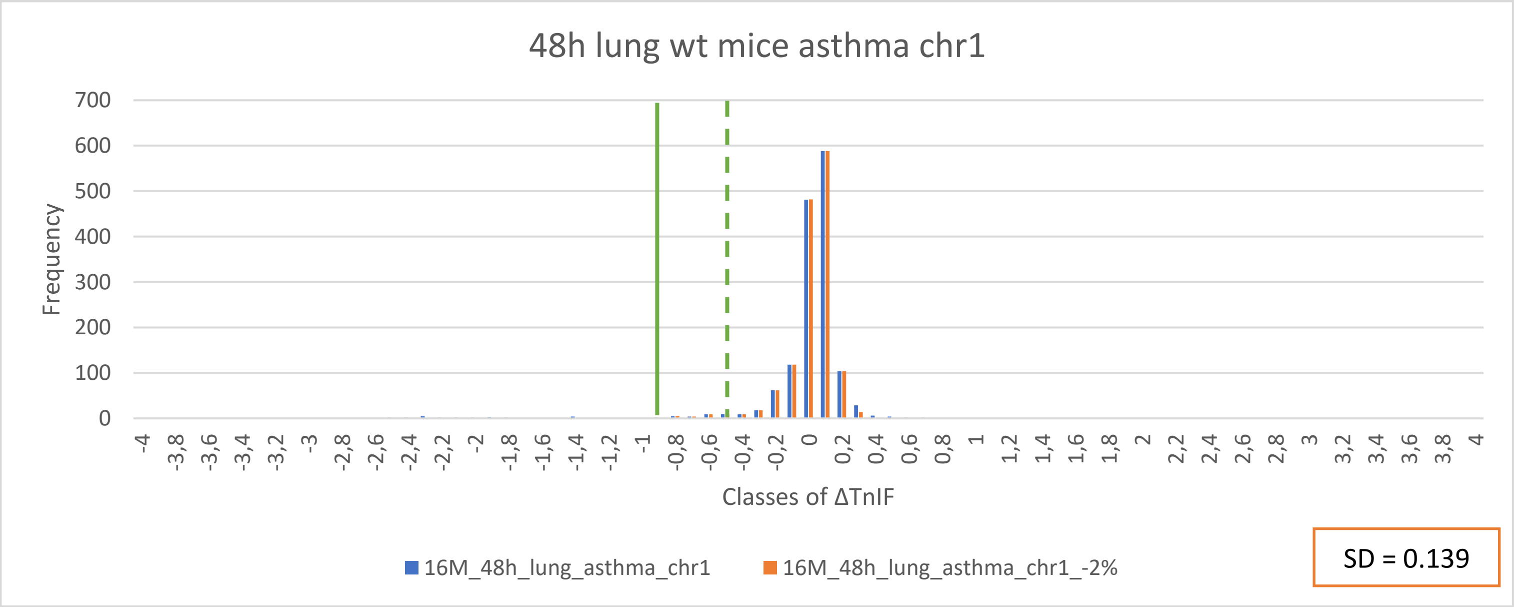
48h lung wt mice asthma chr1
Frequency
700
600
500
400
300
200
100
0
-4 -3,8 -3,6 -3,4 -3,2 -3 -2,8 -2,6 -2,4 -2,2 -2 -1,8 -1,6 -1,4 -1,2 -1 -0,8 -0,6 -0,4 -0,2 0 0,2 0,4 0,6 0,8 1 1,2 1,4 1,6 1,8 2 2,2 2,4 2,6 2,8 3 3,2 3,4 3,6 3,8 4
Classes of ΔTnIF
16M_48h_lung_asthma_chr1
16M_48h_lung_asthma_chr1_-2%
SD = 0.139

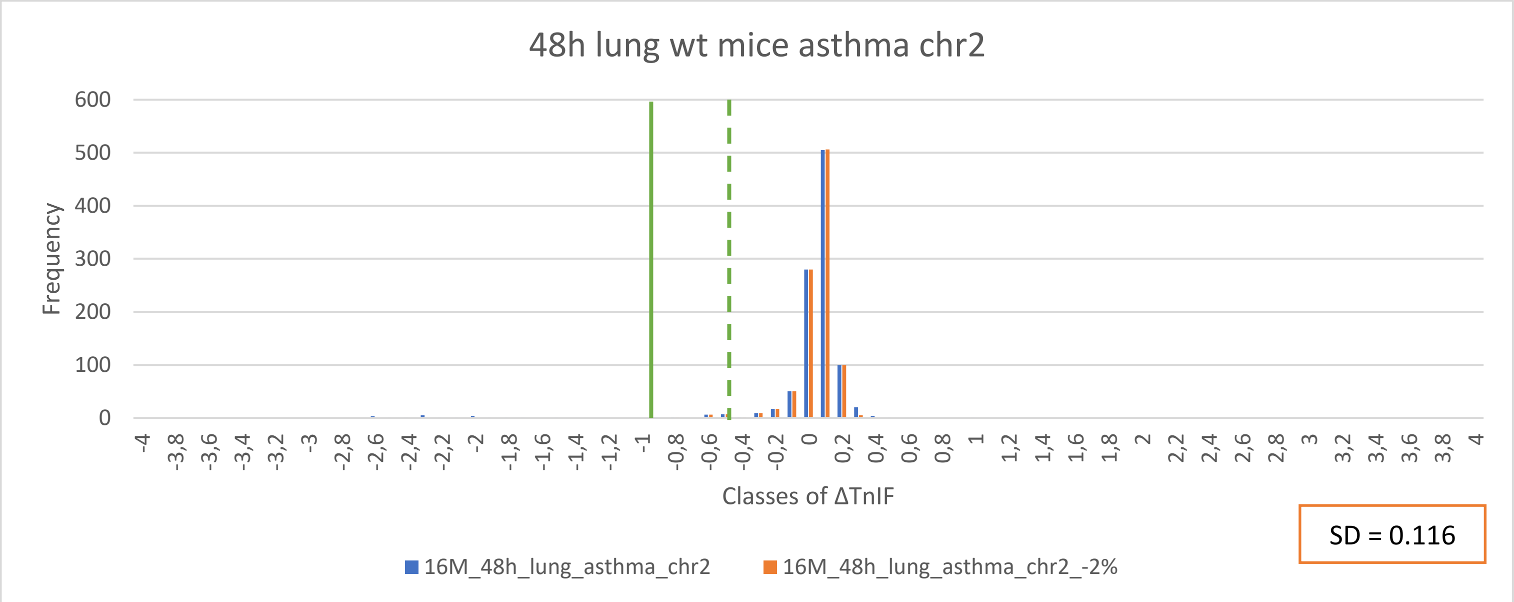

48h lung wt mice asthma chr2
Frequency
Classes of ΔTnIF
16M_48h_lung_asthma_chr2
16M_48h_lung_asthma_chr2_-2%
SD = 0.116

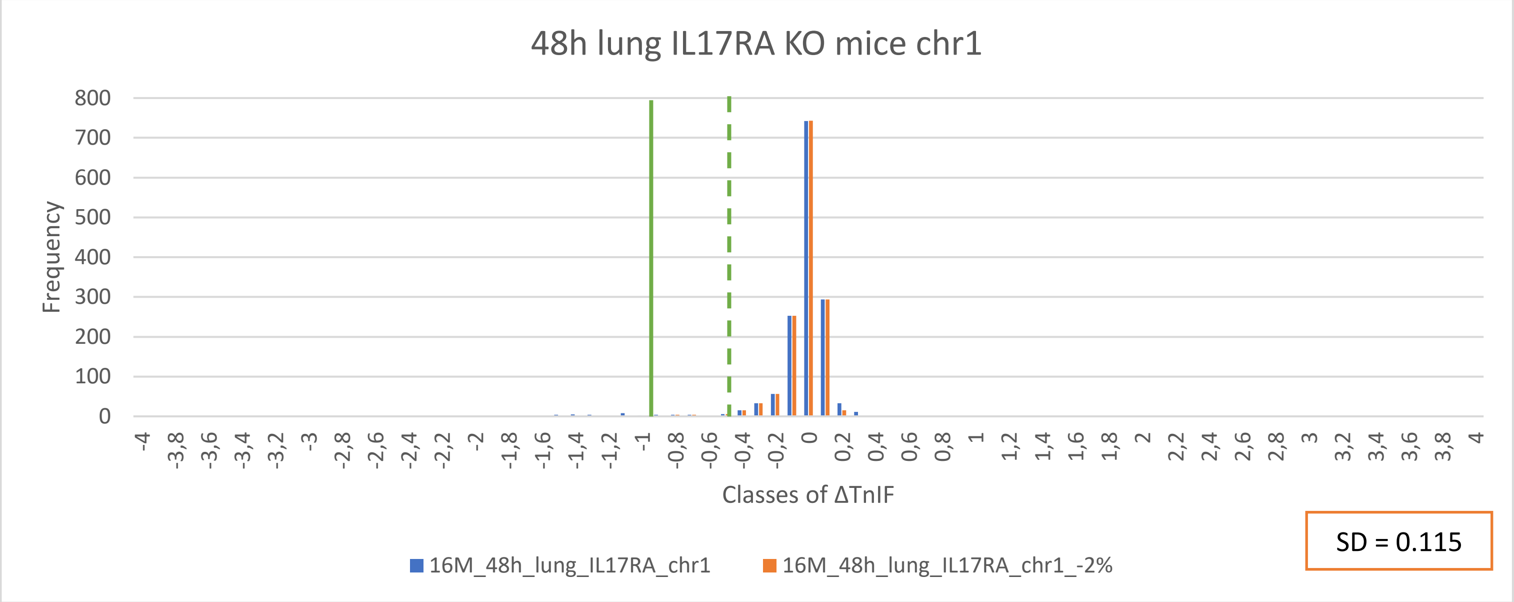

48h lung IL17RA KO mice chr1
Frequency
Classes of ΔTnIF
16M_48h_lung_IL17RA_chr1
16M_48h_lung_IL17RA_chr1_-2%
SD = 0.115

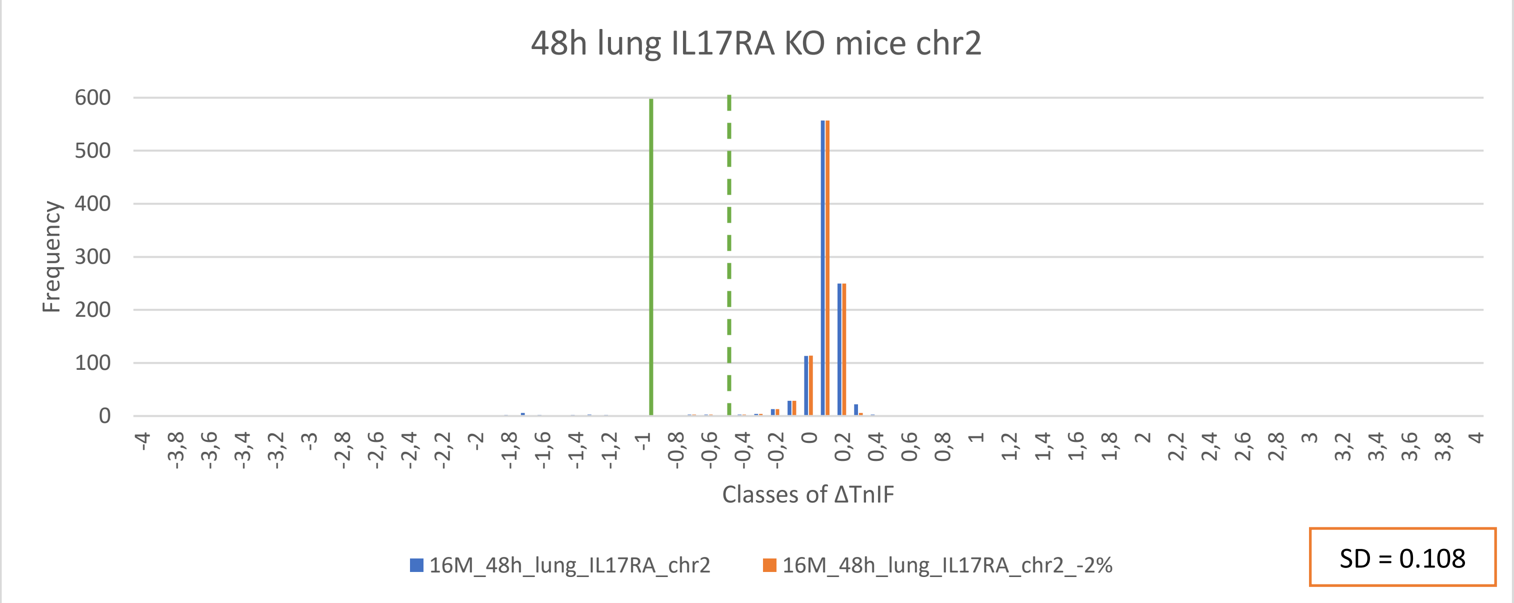

48h lung IL17RA KO mice chr2
Frequency
Classes of ΔTnIF
16M_48h_lung_IL17RA_chr2
16M_48h_lung_IL17RA_chr2_-2%
SD = 0.108

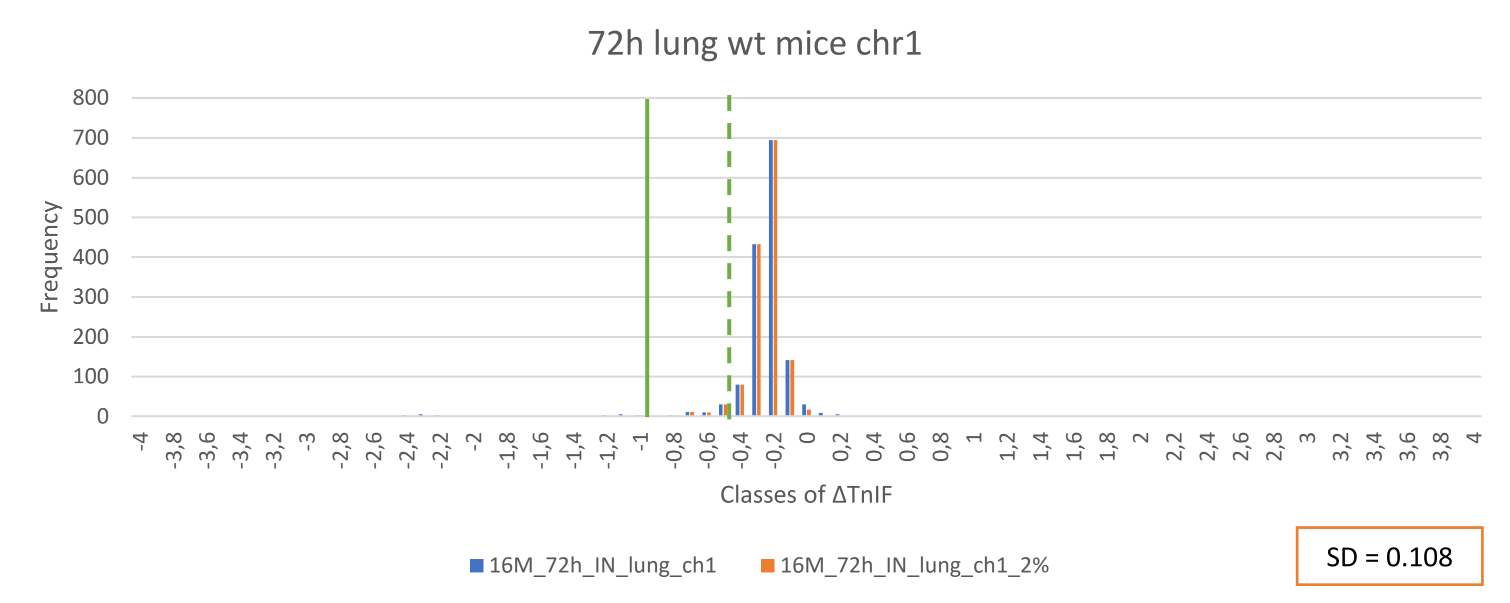

72h lung wt mice chr1
Frequency
Classes of ΔTnIF
16M_72h_IN_lung_ch1
16M_72h_IN_lung_ch1_2%
SD = 0.108

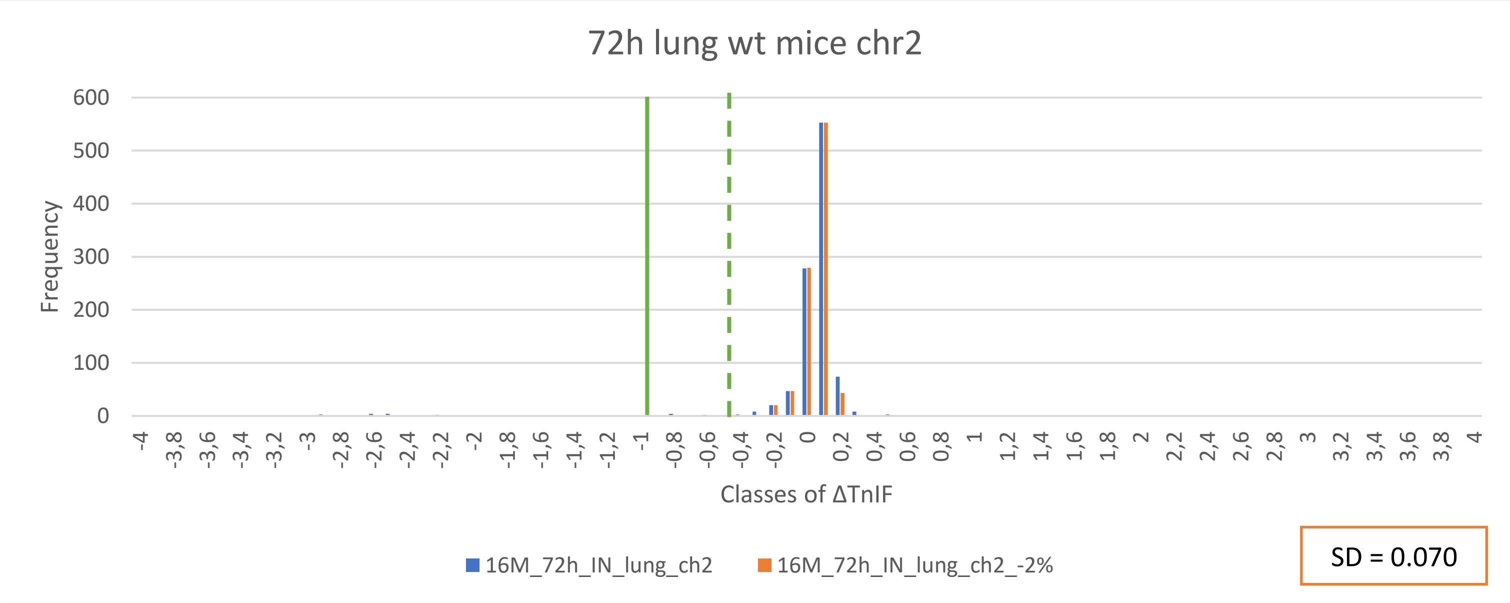

72h lung wt mice chr2
Frequency
Classes of ΔTnIF
16M_72h_IN_lung_ch2
16M_72h_IN_lung_ch2_-2%
SD = 0.070

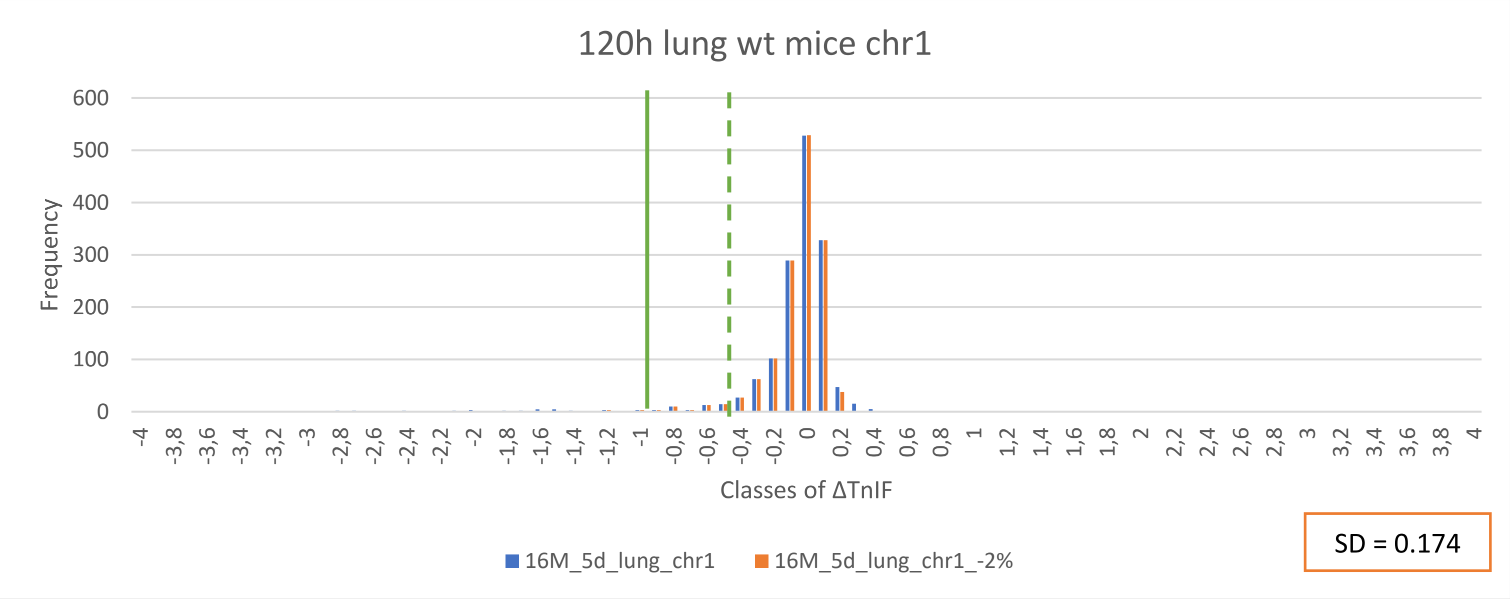

120h lung wt mice chr1
Frequency
Classes of ΔTnIF
16M_5d_lung_chr1
16M_5d_lung_chr1_-2%
SD = 0.174

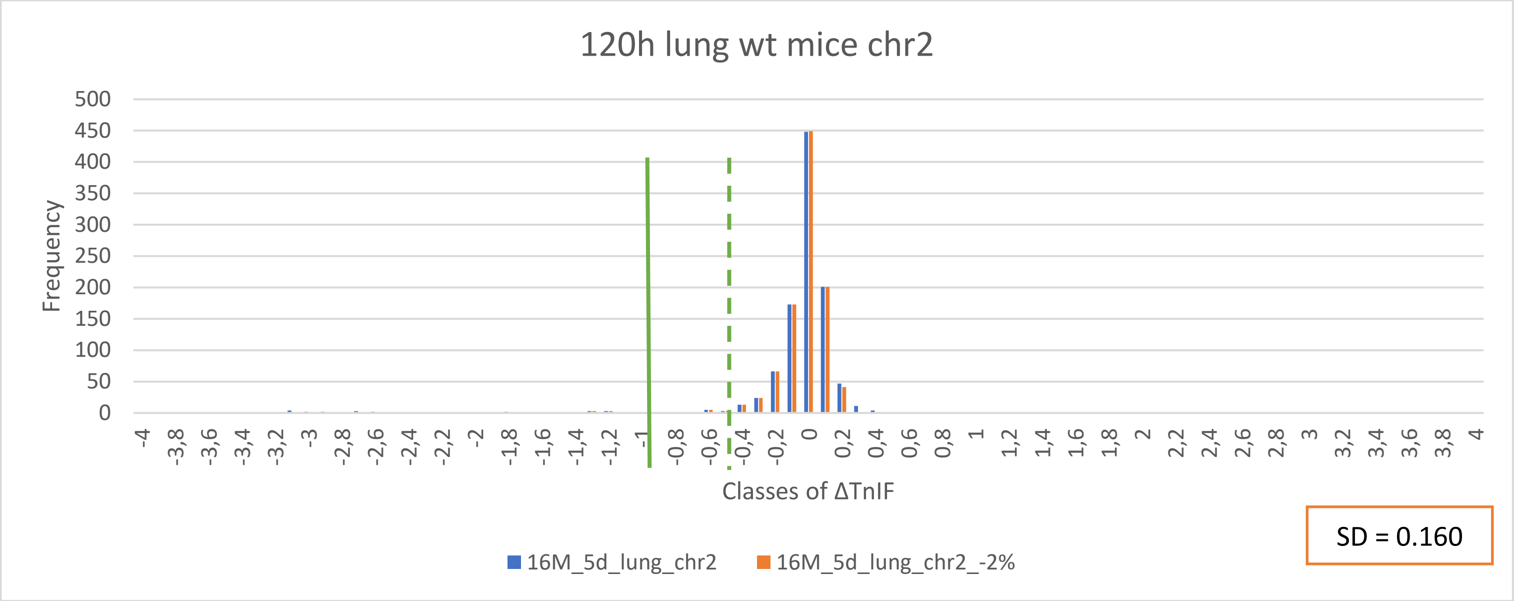
120h lung wt mice chr2
Frequency
500
450
400
350
300
250
200
150
100
50
0
-4
-3,8
-3,6
-3,4
-3,2
-3
-2,8
-2,6
-2,4
-2,2
-2
-1,8
-1,6
-1,4
-1,2
-1
-0,8
-0,6
-0,4
-0,2
0
0,2
0,4
0,6
0,8
1
1,2
1,4
1,6
1,8
2
2,2
2,4
2,6
2,8
3
3,2
3,4
3,6
3,8
4
Classes of ΔTnIF
SD = 0.160
16M_5d_lung_chr2
16M_5d_lung_chr2_-2%
