# Supplementary material for: Genome-wide analysis of Brucella melitensis genes required throughout intranasal infection in mice
Source: PLoS Pathog. 2022 Jun 30;18(6):e1010621. doi: 10.1371/journal.ppat.1010621 (PMC9246152; doi:10.1371/journal.ppat.1010621)
Supplement: S3 Table — (DOCX) [file ppat.1010621.s010.docx]

**Supplementary Table 3: List of primers used in the construction of deletion mutants**

| **BMEII0497 *fadJ*** |  |
| --- | --- |
| Forward_check_*fadJ* | ACCTGATTGCCACCAAATACG |
| Reverse_check_*fadJ* | AGATGCGATTGGGATGCTC |
| Forward_upstream_*fadJ* | GATGATGTCGATGCCTATGCT |
| Reverse_upstream_*fadJ* | AACGAGAGCAATGCCGTC |
| Forward_downstream_*fadJ* | GACGGCATTGCTCTCGTTCCACCAACCCATCAAGCG |
| Reverse_downstream_*fadJ* | GTGCAGTATCTTGGCCATCAC |
| **BMEI0844 *trpD*** |  |
| Forward_check_*trpD* | GCTCGCCACCAATATTGC |
| Reverse_check_*trpD* | CGTCATGTTGTCGCATAAGG |
| Forward_upstream_*trpD* | CTGCACGATTCCTATGAGCAG |
| Reverse_upstream_*trpD* | CACATCCACCGCTTTTAGAAG |
| Forward_downstream_*trpD* | CTTCTAAAAGCGGTGGATGTGGTCATTGCCGTTTCCAACGAC |
| Reverse_downstream_*trpD* | GTCCTCATGCGTGAAGATGC |
| **BMEII0496 *fadA*** |  |
| Forward_check_*fadA* | ATGAATATCCGCGCTTTCAAC |
| Reverse_check_*fadA* | CGACTTCAAAATGGCGAGTG |
| Forward_upstream_*fadA* | TGCGCTTATCCTGTGGATTGC |
| Reverse_upstream_*fadA* | AGCCATGTTTGCTCCTTCCA |
| Forward_downstream_*fadA* | TGGAAGGAGCAAACATGGCTCGGGTGTAAGGGGAGGTTCAG |
| Reverse_downstream_*fadA* | GATGTTTCGCGACGCAGGATG |
| **BMEI1413 *gmd*** | |
| Forward_check_*gmd* | AGCTAACTTGCTGGCATAAG |
| Reverse_check_*gmd* | CAGGTCGAGTCGATCATG |
| Forward_upstream_*gmd_*SpeI | CTAGAACTAGTGTCATTCTGTAATTATACGCAAGC |
| Reverse_upstream_*gmd* | AAGGAATGATCCATACATGG |
| Forward_downstream_*gmd* | CCATGTATGGATCATTCCTTTTTTTGCCATGCCAACTG |
| Reverse_downstream_*gmd_*SalI | TGCACGTCGACCGAAGCGGTTGTAACAGG |
| **BMEI1426 *wbkf*** | |
| Forward_check_*wbkF* | TATTCCTTTGGCTGACCAGCGA |
| Reverse_check_*wbkF* | GTTTGCCTCTTGCACGGTGG |
| Forward_upstream_*wbkF* | GTCGACGTTGGAAGCAAGGGCG |
| Reverse_upstream_*wbkF* | GTGTGGCAACAATTCCGCAGAGAAGGGC |
| Forward_downstream_*wbkF* | CTGCGGAATTGTTGCCACACTCATTCTGCT |
| Reverse_downstream_*wbkF* | ACTAGTGCATTTTCAACAATGTATTCGG |
| **BMEI1414 *per*** | |
| Forward_check_*per* | GAGTATACTGCGAACTCAGAC |
| Reverse_check_*per* | GGTAATCGATGGCTGGAT |
| Forward_upstream_*per* | GTCGACCCACCTCTGAGCTTTATGGTCT |
| Reverse_upstream_*per* | CCAAATCACACTTTTTGAAATTCCCACAGAGATAG |
| Forward_downstream_*per* | TTTCAAAAAGTGTGATTTGGTCAGGAGTTATTTTT |
| Reverse_downstream_*per* | CTGCAGTGGTAGCCGTAGGCCATT |
